# Local and systemic effects of microbiome‐derived metabolites

**DOI:** 10.15252/embr.202255664

**Published:** 2022-08-29

**Authors:** Igor Spivak, Leviel Fluhr, Eran Elinav

**Affiliations:** ^1^ Systems Immunology Department Weizmann Institute of Science Rehovot Israel; ^2^ Medical Clinic III University Hospital Aachen Aachen Germany; ^3^ Microbiome & Cancer Division, DKFZ Heidelberg Germany

**Keywords:** commensals, immune, metabolites, microbiome, postbiotic, Metabolism, Microbiology, Virology & Host Pathogen Interaction, Signal Transduction

## Abstract

Commensal microbes form distinct ecosystems within their mammalian hosts, collectively termed microbiomes. These indigenous microbial communities broadly expand the genomic and functional repertoire of their host and contribute to the formation of a “meta‐organism.” Importantly, microbiomes exert numerous biochemical reactions synthesizing or modifying multiple bioactive small molecules termed metabolites, which impact their host's physiology in a variety of contexts. Identifying and understanding molecular mechanisms of metabolite–host interactions, and how their disrupted signaling can contribute to diseases, may enable their therapeutic application, a modality termed “postbiotic” therapy. In this review, we highlight key examples of effects of bioactive microbe‐associated metabolites on local, systemic, and immune environments, and discuss how these may impact mammalian physiology and associated disorders. We outline the challenges and perspectives in understanding the potential activity and function of this plethora of microbially associated small molecules as well as possibilities to harness them toward the promotion of personalized precision therapeutic interventions.

## Graphical abstract

Microbiomes synthesize or modify multiple bioactive small molecules, which impact their host's physiology. This review highlights key effects of bioactive microbe‐associated metabolites and discusses how they may impact mammalian physiology and associated disorders.

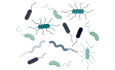

## Introduction

Commensal microorganism consortia, collectively termed the microbiome, inhabit multiple mucosal sites of their mammalian host, and significantly contribute to the “holobiont” in cell numbers and genome size (Tierney *et al*, 2019). The microbiome carries out multiple roles, such as outcompeting pathogens in their habitat, providing signals of homeostatic or perturbed surrounding conditions, and producing, modulating, and degrading a wide range of small soluble bioactive molecules (herein referred to as metabolites, see Table 1), which feature multiple effects on the host. Microbial metabolism often involves the utilization of components not accessible to host metabolic enzymes, such as complex carbohydrates, but can also compete with host metabolic systems for substrates. Microbial products and processes are highly interconnected with the host's own metabolic function in contributing to the host's physiology and homeostasis (Thaiss *et al*, 2016b), complementing the host in degrading complex molecules (Cani *et al*, 2019), sensing different conditions in their environment, and in the regulation of commensal communities (Krautkramer *et al*, 2021). The microbial‐produced metabolite repertoire is dependent on host genetic and ecological diversity (Costello *et al*, 2012; Goodrich *et al*, 2014), geographic location (Hehemann *et al*, 2010), age (Yatsunenko *et al*, 2012; Sato *et al*, 2021), diet (Kolodziejczyk *et al*, 2019; Alexander & Turnbaugh, 2020), and other lifestyle‐related factors (Bajaj, 2019). Certain classes of bacterial metabolites, including short‐chain fatty acids (SCFAs) and bile acids are mostly linked to beneficial effects on their host's health (Koh & Bäckhed, 2020). Different end‐products of microbial tryptophan metabolism can have either a beneficial or harmful impact (Agus *et al*, 2018; Paeslack *et al*, 2022). Others, such as some amino acid derivates or trimethylamine‐oxide (TMAO) are largely associated with noxious effects contributing to disease pathogenesis (Agus *et al*, 2021). Insights into the mechanism of action and modulation of these microbial products may enable their integration as therapeutics (Chaluvadi *et al*, 2016), a modality recently termed postbiotic treatment (Aguilar‐Toalá *et al*, 2018). A recent consensus statement by the International Scientific Association of Probiotics and Prebiotics (ISAPP) proposed to integrate supplementation with inactivated microorganisms (Salminen *et al*, 2021) into this term. However, this inclusive, industry‐backed definition remains highly controversial (Aguilar‐Toalá *et al*, 2021), with many microbiome researchers relating to “postbiotic” therapy only in the context of a well‐defined and evidence‐based small molecule intervention (Cullin *et al*, 2021; Vrzáčková *et al*, 2021; Box 1).

**Table 1:** Different classes of microbial products, disease contexts, and organismal platforms of conducted studies.

Category	Molecule	Source(s)	Platforms of study	Disease context
Short chain fatty acids (SCFAs)	Butyrate, propionate, acetate	Carbohydrates (diet)	*In vitro*, rodents, human: microbial transfer, metabolite supplementation	Intestinal inflammation, metabolic syndrome, adiposity, hypertension, atherosclerosis, ischemic stroke, chronic kidney disease, type‐1 Diabetes mellitus
Carboxylic acid intermediates	Lactate, succinate	Carbohydrates (diet)	*In vitro*, rodents, human: microbial transfer	Metabolic syndrome, intestinal epithelial regeneration, bacterial vaginosis
Amino acids and derivatives	Branched‐chain amino acids (BCAAs), niacin/nicotinamide, 5‐aminovaleric acid, dimethylglycine, acetylglycine	Amino acids (diet and microbial de‐novo synthesis)	*In vitro*, rodents, human: observational	Adiposity, cardiovascular events, amyotrophic lateral sclerosis (ALS), intestinal inflammation and carcinogenesis, atherosclerosis, myocardial infarction, stroke
	Taurine	Primary bile acids (host metabolism)	*In vitro*, rodents	Intestinal inflammation
	Trimethylamine‐oxide (TMAO)	Host metabolism of microbial trimethylamine (TMA)	*In vitro*, rodents, human: observational	Metabolic syndrome, type‐2 Diabetes mellitus, liver steatosis, atherosclerosis, myocardial infarction, ischemic stroke, thrombosis
Pattern receptor recognition (PRR) ligands	Lipopolysaccharide (LPS), peptidoglycan, lipoteichoic acid (LTA), polysaccharide A, bacterial DNA, secreted microbial proteins	Structural components of microbes	*In vitro*, rodents, human: observational	Intestinal inflammation, infection and carcinogenesis, adiposity, liver steatosis, inflammation and fibrosis, acute liver failure, thrombosis, metabolic syndrome, type‐1 Diabetes mellitus
Tryptophan metabolites	Indole‐3‐propionic acid (I3PA), indole‐3‐aldehyde (IAId), indoxylsulfate (IS), tryptamine	Amino acids (diet)	*In vitro*, rodents, human: observational	Intestinal inflammation, fungal infection, hypertension, chronic kidney disease, type‐1 Diabetes mellitus, atopic dermatitis
Flavonoids	Quercetin, apigenin, naringenin	Polyphenols (diet)	*In vitro*, rodents	Adiposity, intestinal inflammation and infection
Secondary bile acids	lithocholic acid (LCA), deoxycholic acid (DCA) & derivatives	Primary bile acids (host metabolism)	*In vitro*, rodents, human: observational	Liver steatosis, inflammation and fibrosis, metabolic syndrome, adiposity, colon cancer, malabsorption and micro‐nutrient deficiency, *C. difficile* infection
Other classes	Tetrahydrobiopterin (BH4), folate, sphingolipids, amyloids	Microbial de‐novo synthesis, amino acids (diet)	*In vitro*, rodents	Metabolic syndrome, intestinal inflammation, Parkinson's disease, autism spectrum disorder (ASD)

ALS, amyotrophic lateral sclerosis; ASD, autism spectrum disorder; BCAAs, branched‐chain amino acids; BH4, tetrahydrobiopterin; DCA, deoxycholic acid; I3PA, indole‐3‐propionic acid; IAId, indole‐3‐aldehyde; IS, indoxylsulfate; LCA, lithocholic acid; LPS, lipopolysaccharide; LTA, lipoteichoic acid; SCFA, short chain fatty acids; TMA, trimethylamine, TMAO, trimethylamine‐oxide.

Box 1 **In need of answers**

Will consensus be reached on a clear definition and classification of therapeutic products derived from microbes or targeting microbial metabolism?

Is the role of certain microbial metabolites in health and disease correlative or causative?

Are the current analytical pipelines for microbiomes and their metabolites reflecting the *in vivo* conditions and can they be reproduced across studies?

What are the translational limitations of microbial metabolite focused studies in animal models to the human setting?

What host factors contribute to beneficial or deleterious effects of microbial metabolites?

Are personalized approaches to make use of the effects of microbial metabolites for health benefits superior to resource‐saving one‐for‐all strategies?

In this review, we aim to exemplify major mechanisms of action of microbially modulated metabolites, impacting host physiology and disease both locally at their site of production and following systemic distribution. Rather than focusing on the description of metabolite classes and their impact on selected organs (addressed in excellent reviews (Wahlström *et al*, 2016; Borghi *et al*, 2020; van der Hee & Wells, 2021)), we illuminate the differential roles played by key microbial metabolites in their local environment of production, as compared to remote effects mediated by metabolite influx into the host's systemic circulation.

## Effects of microbial metabolites on their local environment

**Figure 1:**
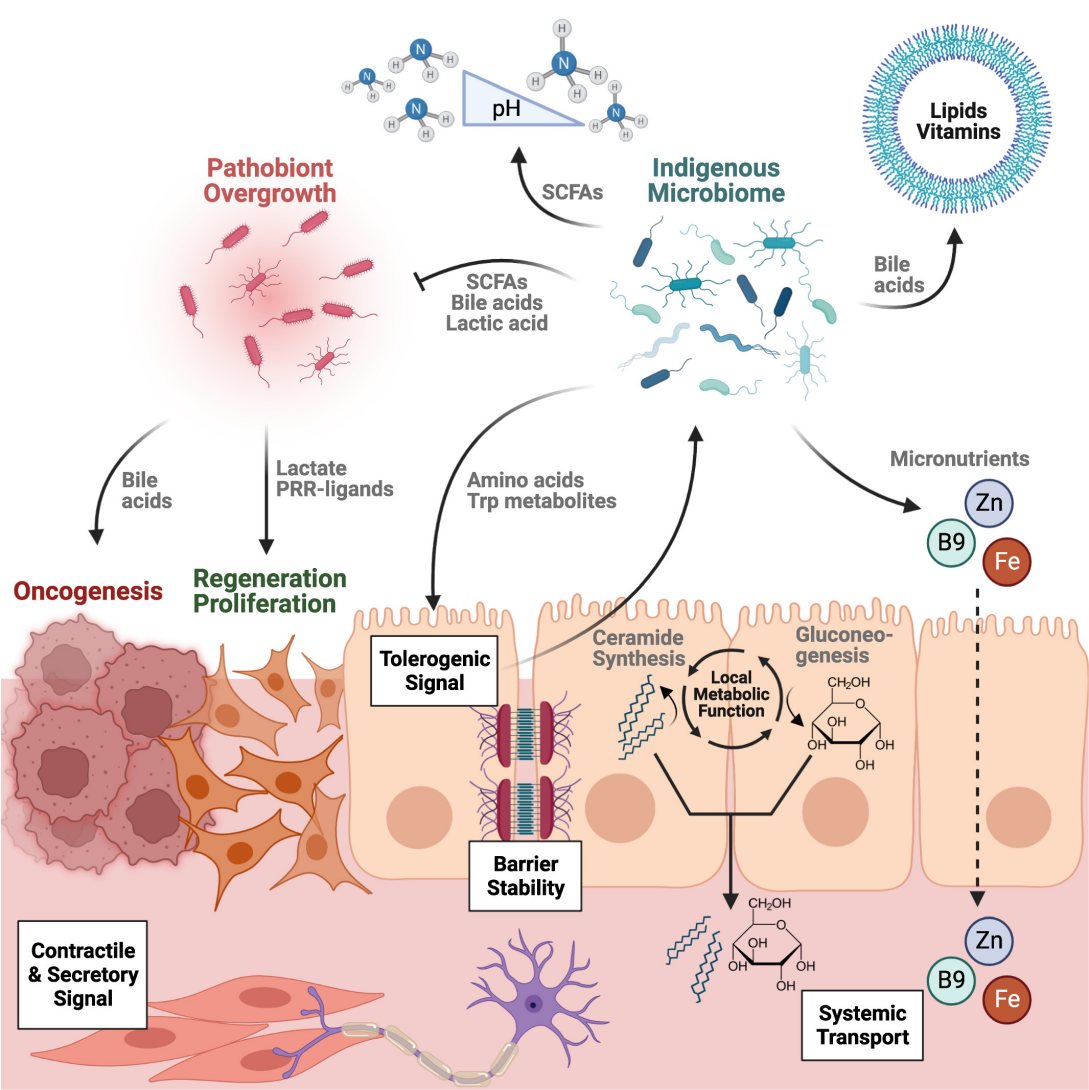
Molecules of microbial origin interact with the local environment at their site of production The indigenous microbiome secretes amino acids and tryptophan metabolites maintaining its own stability through tolerogenic signals received from the host's epithelium. Other commensal factors such as bile acids, lactic acid, or short chain fatty acids (SCFAs) can counteract the overgrowth of pathobionts. Besides supplying micronutrients including vitamin B9, iron, and zinc directly to the host, the microbiome also modifies the absorption of dietary components in the gastrointestinal tract: conjugated bile acids increase the solubility of lipids, while lactic acid contributes to maintain pH levels preventing the absorption of toxic ammonia. Bacterial products such as SCFAs, flavonoids, tryptophan metabolites, amino acids, and toll‐like receptor (TLR)‐ligands contribute to fortify the intestinal barrier, preventing bacteria from dislocating into deeper mucosal layers. Intestinal epithelial cells (IECs) utilize SCFAs as their main metabolic precursors for the harvest of nutrients. They also utilize SCFAs and bile acids in the regulation of metabolic circuits resulting in the production and systemic deployment of glucose or ceramides. SCFAs or tryptamine can promote intestinal motility activating feedback‐loops involving the epithelium, local neurons, and smooth muscles. While lactate and TLR‐agonists can progress regenerative processes of the epithelium after injury, bile acids have the potential to drive proliferative signals facilitating tumor development. SCFA, short chain fatty acid; TLR, toll‐like receptor; Trp, tryptophan. (Created with BioRender.com).

As metabolically active organisms, microbes and their immediate neighboring host tissues have co‐evolved to take advantage of each other's unique metabolic capabilities. Metabolites secreted and modulated by both constituents of the “holobiont” are used in various fashions, ranging from energy sources, sensing of nutrients, directing physiologic responses or preventing the expansion of malicious microorganisms (Fig 1). At times, detrimental processes such as invasive pathogen infections or neoplasia may “hijack” metabolites and their signaling pathways in conferring a competitive advantage to themselves. Key examples of local metabolite functions are depicted below.

### Intestinal barrier function

Microbes inhabit large mucosal surfaces within their host. While commensals contribute to host barrier function in preventing colonization and translocation of exogenous pathogens, the large commensal biomass itself must be regulated by the host barrier in preventing harmful microbial invasion. The microbiome also provides crucial stimuli to the host to maintain the function of this important barrier. For example, tight junctions (TJ) and their cytoskeleton anchors constitute an important component of the intestinal epithelial barrier. Upregulation of their production can be induced by various gut bacteria‐induced metabolites in mouse models, such as SCFAs (Wang *et al*, 2020), products of tryptophan‐degradation, like indole‐3‐propionic acid (I3PA; Venkatesh *et al*, 2014), the flavonoid quercetin (Suzuki & Hara, 2009; Carrasco‐Pozo *et al*, 2013), and amino acids (Grosheva *et al*, 2020). Metabolite‐induced fortification of gut barrier function can increase intestinal resistance to several noxious events modeled in rodents, such as inflammation (Wang *et al*, 2020) or drug‐induced injury (Singh *et al*, 2017). Bacterial toll‐like receptor (TLR)‐ligands activate Nod‐like receptor pyrin domain‐containing protein 6 (NLRP6) inflammasomes in “sentinel” goblet cells (senGCs) in lower crypts of the colon. This triggers mucus secretion and luminal expulsion of senGCs, both fortifying the mucus layer to prevent bacteria from breaching the intestinal barrier (Birchenough *et al*, 2016). The entry of microbes into the systemic circulation is also limited by the gut vascular barrier (GVB; Spadoni *et al*, 2015). Bile acids that are agonists of the farnesoid X receptor (FXR) have a stabilizing effect on the GVB in mouse models of liver injury (Sorribas *et al*, 2019). Bile acid deconjugation through gut microbes generally impacts the bile acid pools toward FXR agonists in mice and humans (Tremaroli *et al*, 2015; Parséus *et al*, 2017; Quinn *et al*, 2020) and therefore could play a role in maintaining the GVB. A combinatorial impact of multiple, yet uncharacterized barrier‐strengthening and disrupting metabolites (Grosheva *et al*, 2020) likely contributes to differences in barrier function observed in different clinical and pathological contexts and merits future mechanistic studies.

### Energy source

Microbial metabolites may also serve as sources of energy for their surrounding tissue. For example, as a result of co‐evolution with their neighboring microbiome, intestinal epithelial cells (IECs) have adapted to harness nutrients in a variety of metabolic processes, such as oxidation, lipogenesis or the generation of ketone bodies, and amino acids (Bugaut, 1987; Bergman, 1990). SCFAs generated through microbial carbohydrate fermentation are utilized by IECs as energy sources (den Besten *et al*, 2013a). Additionally, a study in rats found vasoactive intestinal peptide (VIP) inducing epithelial gluconeogenesis, while propionate led to an increase of VIP‐expressing neurons in the gut submucosal plexus (De Vadder *et al*, 2015). SCFAs are also able to engage in epithelial lipid metabolism. Acetate can be transformed into the metabolic intermediate Acetyl‐CoA in IECs. This leads to activation of the anti‐lipogenic master regulator AMP‐activated protein kinase (AMPK) and therefore to increased fatty acid oxidation and downregulated lipid production. As a consequence, fewer lipids are shuttled to the lymph and systemically available as chylomicrons (Araújo *et al*, 2020). Acetyl‐CoA derived from L‐lactate of microbial origin features an opposite effect on lipid metabolism in IECs. Its transformation to Malonyl‐CoA results in intracellular lipogenesis and decreased oxidation of lipids. Surprisingly, this leads to lower secretion of lipids from IECs despite an increased storage (Araújo *et al*, 2020). However, lipogenesis triggered by microbial metabolites can also have undesirable outcomes in downstream organs. Exposure to bile acids that act as FXR‐agonists leads to IEC‐production of ceramides, lipid molecules consisting of a sphingosine and fatty acid, contributing to hepatic lipogenesis in mice (Jiang *et al*, 2015). The beneficiary effect of the antidiabetic drug metformin depleting *B. fragilis* recently reported in a human cohort of Type‐2‐diabetics (Sun *et al*, 2018) could be a result of disruptions of microbial folate and methionine metabolism (Induri *et al*, 2022). Through this mechanism, the ability of *Bacteroides* to transform bile acids that fuel ceramide production is abrogated (Sun *et al*, 2018).

Microbially modulated bile acids can also alter the response of the endocrine pancreas to incretins released from intestinal L‐cells in response to dietary signals. Mouse models found bile acids deconjugated by *Clostridium* and *Bacteroides* to bind the membrane G protein‐coupled bile acid receptor 1 (TGR5) on L‐cells, inducing the release of insulinotropic glucagon‐like peptide 1 (GLP‐1; Pathak *et al*, 2018), possibly through inhibition of local glycolysis (Trabelsi *et al*, 2015). In contrast, bile acid engagement with the nuclear receptor FXR in L‐cells leads to a downregulation of SCFA‐receptors, thereby abrogating their positive effects on GLP‐1 release (Ducastel *et al*, 2020).

### Intestinal motility

Both commensals, their dietary exposures (Dey *et al*, 2015), and microbial metabolite repertoires are involved in gut motility and the control of luminal content. SCFAs are able to trigger the release of the hormone peptide YY (PYY) from L‐cells. As a late postprandial signal, PYY in murine *ex vivo* mucosa led to an inhibition of chloride driving fluid secretion into the gut lumen. It also interacts with both layers of the enteric smooth muscle as well as neurons belonging to the vagal afferent system leading to an antisecretory response and inhibition of gastro‐intestinal motility *in vivo* (Tough *et al*, 2011). SCFAs can also induce pro‐contractile responses. Neurons located in the rat intestinal myenteric plexus bind butyrate through monocarboxylate transporter 2 (MCT2) receptors, resulting in propulsive contractions (Soret *et al*, 2010). The same effect is observed upon acetylcholine release triggered from IECs upon exposure to propionate (Yajima *et al*, 2011). SCFAs are also capable to act on central nervous sensory and sympathetic ganglia in mice through transcriptional regulation, modulating gastrointestinal motility through a gut–brain circuit (Muller *et al*, 2020). A different bacterial metabolite, tryptamine derived from tryptophan by *R. gnavus* and *C. sporogenes*, exerts pro‐contractile signals (Bhattarai *et al*, 2018). Upon binding serotonin‐receptors, adenylate cyclase activation leads to luminal secretion of Cl^−^ and HCO3^−^ ions as well as water, leading to a shortened colonic transit time in humanized mice (Bhattarai *et al*, 2018).

### Intestinal digestion and absorption

Microbial metabolites may modify the digestion and absorption of dietary compounds. Bile acids are responsible for the emulsification of hydrophobic fat droplets into micelles and therefore heavily influence the digestion and absorption of lipids and fat‐soluble vitamins (A, D, E, K). Microbial deconjugation of bile acids tends to reduce their emulsifying potential (Swann *et al*, 2011). However, most of the absorption of dietary lipids and vitamins takes place in the small intestine (Iqbal & Hussain, 2009), while bacterial deconjugation through bile salt hydrolases is commonly found in the large intestine (Guzior & Quinn, 2021). This geographical distinction can drastically change after surgical creation of blind loops or the congenital occurrence of large diverticula in the small intestine. These regions of blind loops, isolated from the regular gut transit, can undergo an overgrowth of anaerobic bacteria capable of bile salt deconjugation. The consequential impaired emulsification can eventually lead to lipid maldigestion causing diarrhea and a deficiency of a number of vitamins due to malabsorption (Quigley *et al*, 2020). Furthermore, microbial‐derived products also feature a regulatory impact on intestinal lipid absorption and digestion, as these processes were found defective in germ‐free mice. As such, bacterial metabolites may trigger the release of enzymes involved in digestion and transepithelial shuttling of lipids (Martinez‐Guryn *et al*, 2018) and the maturation of lymphatic vessels draining lipids absorbed in the intestine (Suh *et al*, 2019). The production of SCFAs from colonic microbial fermentation results in local acidification, which may augment absorption. One example is the shift from ammonia (NH_3_) toward the less diffusable cationic ammonium (NH_4_^+^) under acidic conditions. This principle has been applied for more than half a century in the treatment of hepatic encephalopathy, in which ammonia cannot be detoxified due to poor liver function. Lactulose, a nonabsorbable disaccharide, contributes to acidic conditions upon microbial digestion in the colon and trapping of ammonia (Agostini *et al*, 1972).

Gut commensals are also capable of producing micronutrients. Though these compounds maintain numerous host metabolic, cellular, and immune functions, only few insights exist about the systemic impact of their bacterial synthesis (Biesalski, 2016). A defective gut bacterial production of folic acid was recently suggested to be linked to the development of Parkinson's disease (PD) in a human study cohort (Rosario *et al*, 2021), in line with previous findings of lowered systemic folate levels in patients with PD (dos Santos *et al*, 2009) and PD‐like symptoms in mouse models of folic acid deficiency (Duan *et al*, 2002). The microbial generation of SCFAs through carbohydrate fermentation may impact the intestinal bioavailability and absorption of certain trace elements. The supplementation of dietary fiber leads to an increase of trans‐epithelial iron transport in the cecum in a rat model of anemia (Lobo *et al*, 2014), while improving the bioavailability of zinc in another study (Scholz‐Ahrens & Schrezenmeir, 2002). Nevertheless, the impact of metabolites of microbial origin in determining the bioavailability of trace elements merits further studies, especially in the human setting (Coudray *et al*, 1997).

### Protection against pathogens and pathobionts

Microbial metabolites are involved in the reaction against the overgrowth and invasion of potentially noxious organisms while allowing indigenous commensals to harbor their niches. For example, intestinal infection with *C. difficile* is often triggered by a disruption of eubiotic communities through broad‐spectrum antibiotic treatment. *C. scindens* was found to counteract the blossom of this pathobiont through deconjugation of the bile acid cholic acid (Buffie *et al*, 2015). The augmentation of a healthy intestinal microbial community through fecal microbial transplantation (FMT) is effective as treatment of therapy‐resistant *C. difficile* infection. Metabolites derived from the transplanted microbiome, such as the bile acids deoxycholic acid (DCA) and lithocholic acid (LCA), and the SCFAs propionate and butyrate may contribute to this beneficial effect (Seekatz *et al*, 2018).

Microbial communities can induce tolerogenic signals in their host, thereby enabling the persistence of microbes with beneficial effects and preventing the overgrowth of pathogenic strains. Tryptophan metabolites produced by lactobacilli lead to Il‐22 release upon aryl hydrocarbon receptor (AHR) signaling from intestinal immune cells, counteracting *Candida* colonization through an amplified antifungal immune response (De Luca *et al*, 2010; Zelante *et al*, 2013). Vice versa, Il‐22 secretion also serves to maintain a community rich in bacterial AHR‐ligand producers: Defective Il‐22 signaling in mice led to dysbiosis concomitant with low AHR‐ligand production and colitis susceptibility, while in humans, lower gut microbial AHR activation and a single nucleotide polymorphism linked to impaired Il‐22 function were associated with inflammatory bowel disease (Lamas *et al*, 2016). A similar effect was observed upon gut microbial taurine production in mice inducing epithelial NLRP6 inflammasome activation and Il‐18 secretion. Subsequently, the release of antimicrobial peptides (AMPs) counteracted an overgrowth of bacteria that lead to an inflammatory reaction and sustained the commensal taurine producers (Levy *et al*, 2015).

Beyond the gut, lactic acid produced in the vaginal mucosal surface by resident lactobacilli (most effectively by the *crispatus* species) is present in its protonated form at pH levels < 3.9. In this state, it exerts direct antimicrobial effects protective of bacterial overgrowth (Tachedjian *et al*, 2017), coupled with virucidal effects on HIV and HSV‐2 (Conti *et al*, 2009; Aldunate *et al*, 2013). A recent first‐in‐human clinical trial suggested that restoration of the vaginal microbiome with vaginal microbiome transplantation (VMT) successfully reestablishes lactic acid‐producing communities and reverses the overgrowth of anaerobes, collectively improving intractable and recurrent bacterial vaginosis and its associated symptoms (Lev‐Sagie *et al*, 2019). The contribution of metabolite shifts to this effect merit further studies.

### Epithelial cell replenishment

Epithelial cells are frequently replaced through differentiation and proliferation of intestinal stem cells. This enables intestinal regeneration after injury, but, when excessive and unregulated, also increases susceptibility toward colorectal cancer development. Bacterial‐produced lactate is involved in a number of proliferative processes in the intestinal epithelium. It interacts with Paneth cells through the receptor GPR81, leading to epithelial expansion and maintenance of intestinal stem cells (ISCs) in mice through Wnt/β‐catenin signals (Lee *et al*, 2018). Recognition of bacterial components through specialized pattern recognition receptors (PRRs) plays an important role in all phases of gut epithelial regeneration after injury: In mouse models of chemical colitis it was involved in initial epithelial restitution (Fukata *et al*, 2006; Normand *et al*, 2011), proliferation (Hsu *et al*, 2010; Zaki *et al*, 2010), and eventually differentiation (Podolsky *et al*, 2009; Round *et al*, 2011). Mice subjected to starvation exert a hyperproliferation of IECs upon refeeding triggered by lactate as a metabolite derived from *Lactobacillus murinus* (Okada *et al*, 2013). Additionally, chronic inflammatory or premalignant conditions may trigger proliferative signals from microbial metabolites that can induce oncogenic pathways. For example, intestinal adenoma‐prone Apc^Min^ mouse exposure to deoxycholic acid, a product of bacterial bile acid deconjugation, may trigger the intestinal adenoma–carcinoma pathway concomitant with activation of the inflammatory Il6‐STAT3 axis (Wang *et al*, 2019). Sensing of bacterial components through TLR2 and nucleotide binding and oligomerization domain 2 (NOD2) induces the expression of major histocompatibility complex (MHC) class II in ISCs. This contributed to immune surveillance of the intestinal epithelium and prevented the formation of tumors under premalignant conditions in mice (Beyaz *et al*, 2021).

## Systemic impacts of microbial products on the host

**Figure 2:**
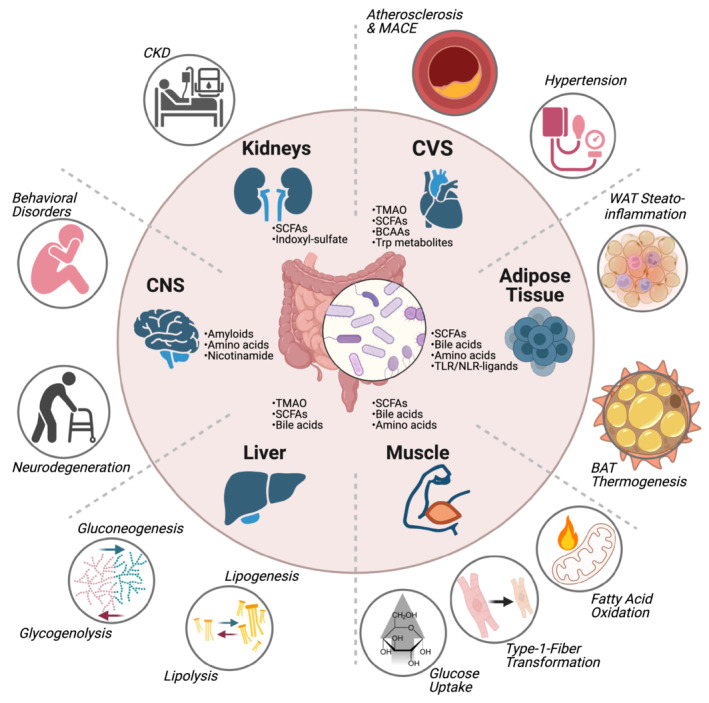
Microbial metabolites systemically impact host physiology Numerous metabolic functions of the host are influenced by products of microbial origin. Short‐chain fatty acids (SCFAs) can act as precursors for lipid or glucose production in the liver. SCFAs and bile acids may also induce hepatic pathways to degrade glucose and lipids. In muscle, SCFAs can increase the net uptake of glucose and induce the transformation to type‐1 fibers capable of increased fatty acid oxidation. Additional improvements of muscle metabolism can be induced by bile acids or amino acids, such as a higher energy expenditure. Adipose tissue may respond to SCFAs or bile acids modulating the transformation of white adipose tissue (WAT) to brown adipose tissue (BAT) to enhance thermogenesis. Microbe‐associated molecular patterns activating toll‐like receptors (TLRs) and nucleotide binding and oligomerization domain receptors (NLRs) as well as microbiome‐modulated amino acids may trigger steato‐inflammation in adipose tissue. Amino acids of bacterial origin, particularly trimethylamine‐oxide (TMAO) and branched‐chain amino acids (BCAAs), are linked to the development of atherosclerosis, myocardial infarction or stroke. In patients suffering from chronic kidney disease (CKD), SCFAs may modulate disease progression, while an impaired degradation of the tryptophan metabolite indoxylsulfate (I3S) could aggravate uremic symptoms. In the central nervous system (CNS), microbial metabolic dysfunction resulting in a lack of nicotinamide in amyotrophic lateral sclerosis (ALS) or an excess of amyloids in Parkinson's disease (PD) contribute to neurodegeneration and worsening motor functions. Behavioral disorders, such as autism spectrum disorder (ASD) are accompanied by alterations of the microbial production of amino acids, such as 5‐aminovaleric acid and taurine upon microbiome transfer to germ‐free mice or tetrahydrobiopterin (BH4) in a mouse model of ASD. ASD, autism spectrum disorder; BAT, brown adipose tissue; BCAA, branched‐chain amino acid; CKD, chronic kidney disease; CNS, central nervous system; CVS, cardiovascular system; MACE, major adverse cardiovascular event; NLR, nucleotide binding and oligomerization domain receptor; SCFA, short chain fatty acid; TLR, toll‐like receptor; TMAO, trimethylamine‐oxide; Trp, tryptophan; WAT: white adipose tissue. (Created with BioRender.com).

In addition to the aforementioned local mechanisms of metabolite activity, their distribution throughout the body by portal and systemic circulation may result in interaction with a variety of remote organs and cells, where they may influence their host's systemic homeostasis and risk of disease (Fig 2, Table 1). In the below section, we exemplify some of these effects by focusing on metabolites impacting systemic features of host metabolism. In a separate section, immune‐mediated effects will be discussed.

### Glucose metabolism

Every cell in the mammalian body is capable of utilizing glucose as a source of energy. Systemically distributed microbial products act as metabolic precursors and in signaling cascades modifying their hosts' glucose metabolism. The potential of SCFAs to act as gluconeogenic substrates through incorporation into the tricarboxylic acid (TCA) cycle has already been mentioned in the previous local metabolic section. Systemically, radiolabeling suggests that propionate may also contribute to a net glucose synthesis (den Besten *et al*, 2013b). The intestinal metabolic capacity significantly contributes to energy homeostasis, with SCFAs converted to glucose in IECs inducing decreased bodyweight, improved insulin sensitivity, and ameliorated hepatic glycogenolysis in mice (De Vadder *et al*, 2014, 2016). Propionate interacting with periportal afferent neurons was suggested to drive this effect (De Vadder *et al*, 2014). SCFAs can act as precursors of two major energy sources of skeletal muscle, glucose and fatty acids. They are also involved in muscle cell uptake of glucose through an increase of intracellular transport (Yamashita *et al*, 2009). SCFAs can induce a switch toward the fatty‐acid oxidizing type‐I fiber, facilitating glucose clearance and increasing insulin sensitivity in mice (Gao *et al*, 2009).

Additionally, microbially deconjugated secondary bile acids have beneficial metabolic effects on skeletal muscles. In a mouse model, TGR5‐triggered cAMP‐dependent thyroid hormone‐activated enzyme type 2 iodothyronine deiodinase (D2), thereby leading to a higher energy expenditure (Watanabe *et al*, 2006). Amino‐acid derivatives undergo metabolic conversions through both host and microbial enzymes. Trimethylamine‐oxide (TMAO) is generated in the liver from trimethylamine, which is produced by gut microbes from multiple precursors including choline, carnitine, and betaine (Chhibber‐Goel *et al*, 2016). In rodents, TMAO may modulate glucose homeostasis via induction of the unfolded protein response (UPR), though the downstream results remain debated. Work by Dumas *et al* (2017) suggests that TMAO induces an improved insulin secretion and glucose tolerance in mouse models of metabolic dysfunction, while others (Chen *et al*, 2019b) argue that TMAO interacts with the UPR‐effector PERK, thereby disrupting glucose tolerance. A study in non‐diabetic humans did not find serum levels of TMAO to be associated with fasting glucose or insulin resistance, but suggested that it may constitute an independent predictor of meeting one of the prediabetes‐defining criteria (Roy *et al*, 2020).

### Adiposity

Historic observations in human subjects suggested a correlation between plasma SCFA levels and adipose tissue mass (Björntorp & Hood, 1966). In agreement, a study comparing gut microbial community structure in lean and obese twins (Turnbaugh *et al*, 2009), and another utilizing FMT from obese donors or those successfully reducing weight following bariatric surgery (Tremaroli *et al*, 2015) concluded that gut microbes from obese subjects harbor the capacity to harvest energy through enhanced carbohydrate fermentation and generation of SCFAs. FMT from lean and obese human twins into germ‐free mice demonstrated a decline in adiposity in recipients of microbiomes from lean individuals, accompanied by an increased capacity to metabolize carbohydrates and higher propionate and butyrate levels (Ridaura *et al*, 2013). In contrast, human FMT from lean to obese subjects failed to alter SCFA levels (Mocanu *et al*, 2021) or induce sustainable metabolic improvements (Zhang *et al*, 2019). Other studies in mice suggested that higher SCFA availability may induce white adipose tissue (WAT) browning and an expansion of beige adipose tissue (BAT; Li *et al*, 2017, 2019; Weitkunat *et al*, 2017), potentially mediated by fatty acid oxidation (Gao *et al*, 2009), phosphorylation of AMPK, or reduction of PPARγ‐signaling (den Besten *et al*, 2015; Gao *et al*, 2019). Besides a direct impact on adipose tissue, SCFAs can cross the blood–brain barrier (Li *et al*, 2019) and modulate neural circuits by releasing neuropeptide Y (NPY) that drives BAT activation and upregulation of thermogenesis (Li *et al*, 2018) in mouse models of obesity. Moreover, the release of the peptide hormone leptin from adipocytes, best known for its effects on satiety, is triggered by SCFAs (Xiong *et al*, 2004; Zaibi *et al*, 2010).

Additionally, microbially modulated bile acids may participate in regulation of adiposity (Tremaroli *et al*, 2015). The exposure of mice to cold temperatures led to changes in the gut microbiome and bile acid profile triggering lipolysis (Ziętak *et al*, 2016; Worthmann *et al*, 2017). Switches in the microbial bile acid metabolic capacity can result in functional reprogramming of the adipose tissue (Li *et al*, 2013; Jiang *et al*, 2015; Pathak *et al*, 2018) enabling thermogenesis, lipolysis, and mitochondrial uncoupling (Broeders *et al*, 2015; Somm *et al*, 2017; Velazquez‐Villegas *et al*, 2018). TMAO (originating from a bacterially produced precursor) may induce or aggravate hepatic steatosis (Chen *et al*, 2016; Tan *et al*, 2019; León‐Mimila *et al*, 2021). A possible mechanism for this effect may involve microbial producers of TMA (the TMAO precursor) outcompeting the host for choline. The hepatic and visceral adipogenesis are a result of impaired DNA‐methylation as well as decreased availability of phosphatidylcholine for lipoprotein synthesis (Romano *et al*, 2017).

In other contexts, dysbiosis in cigarette‐smoking humans and smoke‐exposed mice may increase the microbial conversion of the amino acid glycine into betaine and subsequently dimethylglycine (DMG), concurrently depleting acetylglycine (ACG). Upon smoking cessation, the dysbiosis in mice remained persistent, while the active smoking‐related anorexigenic molecules dissipated, with a combination of increased DMG and depleted ACG contributing to the exacerbation of smoking cessation‐related weight gain mediated through increased energy harvest. Interestingly, these metabolite effects may also be active upon supplementation to non‐smoking obese mice (Fluhr *et al*, 2021). Another common and poorly understood obesity pattern in humans, involves recurring and gradually exacerbating cycles of obesity, also termed “yoyo” obesity. In mouse models of this obesity pattern, apigenin and naringenin, flavonoids modulated by the gut microbiome, are depleted upon induction of obesity, but fail to replenish upon successful dieting. Depletion of these flavonoids contributes to a susceptibility to excessive weight regain upon exposure to further bouts of obesity by impaired regulation of BAT decoupling of oxidation‐phosphorylation, driving excessive net fat accumulation (Thaiss *et al*, 2016a).

### Lipid metabolism

Hyperlipidemia, a component of the metabolic syndrome and a contributor to cardiovascular morbidity, may likewise be influenced by the microbiome (Zmora *et al*, 2018). Several metabolites may impact the metabolism of lipids at sites distant from their production. The liver, exposed to an array of metabolites originating from the gut through the portal vein, exerts a key role in lipid metabolism. The SCFAs butyrate and acetate supplemented to mice were shuttled from the gut and served as precursors for palmitate and cholesterol synthesis in the liver (den Besten *et al*, 2013b). Whether this function additionally contributes to obesity (Samuel *et al*, 2008) or fatty liver in humans (Chambers *et al*, 2019) remains debatable. This is due to the fact that numerous other studies found SCFAs potentially modulating lipid levels through several mechanisms including an activation of the master regulator AMPK/SREBP‐1 axis (Gao *et al*, 2009; den Besten *et al*, 2015; Wu *et al*, 2019), increased β‐oxidation of fatty acids (Gao *et al*, 2009; den Besten *et al*, 2015), and long‐chain fatty acid (LCFA)‐induced modulation of the ω6/ω3 ratio (Weitkunat *et al*, 2016). Sphingolipids (SL) produced by gut bacteria downregulate the host production of SLs in the liver (Johnson *et al*, 2020), a process linked to metabolic disorders such as insulin resistance and nonalcoholic steatohepatitis (Apostolopoulou *et al*, 2018).

### Cardiovascular system

Cardiovascular disease (CVD) complications, including myocardial infarction (MI) or stroke, constitute a major source of morbidity and mortality in individuals suffering from the above features of the metabolic syndrome (Alberti *et al*, 2006; Roth *et al*, 2020). In the past decade, the microbiome and its metabolites were suggested to contribute to the pathogenesis of CVD. For example, gut microbiomes of omnivorous people harbored a higher capacity of processing L‐carnitine compared to those of vegetarians and vegans, leading to increased systemic levels of TMAO (Koeth *et al*, 2013). Higher plasma L‐carnitine and TMAO levels were identified as risk factors for coronary atherosclerosis and major cardiovascular events (Koeth *et al*, 2013; Senthong *et al*, 2016). Increased levels of TMAO were also associated with higher risk of stroke in the presence of hypertension or atrial fibrillation (Nie *et al*, 2018; Liang *et al*, 2019) A possible mechanistic explanation of this increased risk (Zhu *et al*, 2016) included a direct impact of TMAO on the activation of platelets, a critical step in the emergence of clot formation driving ischemic cardiovascular events. As such, FMT of gut microbial communities featuring enhanced capability of TMAO production to humanized mice induced a pro‐thrombotic vascular dysfunction (Zhu *et al*, 2016). Structural components of bacteria can influence clot formation through PRR‐interaction: Platelet TLR2 was found to mediate aggregation *in vitro* upon *S. pneumoniae* recognition (Keane *et al*, 2010), while in mice, LPS triggered platelet TLR4 expression (Aslam *et al*, 2006) resulting in adhesion and accumulation in the lung capillaries (Andonegui *et al*, 2005). Sensing bacterial signals through NOD‐receptors on platelets induced MAPK and NO‐signaling pathways eventually increasing clot formation in mesenteric vessels in a mouse model of thrombosis (Zhang *et al*, 2015). Branched‐chain amino acids (BCAAs) were also linked to cardiovascular events like MI or stroke, although this effect may have been indirectly driven by impaired glucose metabolism (Tobias *et al*, 2018). Humans with acute ischemic stroke were found to harbor alterations of gut microbial communities associated with decreased SCFA production (Tan *et al*, 2021). In rats that developed similar dysbiotic traits upon ischemic stroke, replenishment of SCFA levels through FMT or direct butyrate supplementation led to improved neurological outcomes (Chen *et al*, 2019a). Another small study (Hayashi *et al*, 2021b) suggested that gut microbial community alterations were linked with an enhanced BCAA synthesis in people suffering from heart failure. Interestingly, tryptophan derivatives of bacterial and host origin may impact cardiovascular health in opposing manners. AHR‐agonists of bacterial origin like indole‐3‐acetic acid (IAA) alleviated hypertension in mice (Wilck *et al*, 2017), while host production of kynurenines from tryptophan was associated with acute coronary events in a study cohort of elderly humans (Eussen *et al*, 2015). Microbial metabolites may also prevent atherosclerosis. A genome‐wide association study suggested that microbially produced butyrate is negatively associated with atherosclerotic cardiovascular disease (Jie *et al*, 2017). Likewise, studies in rodents (Marques *et al*, 2017; Wang *et al*, 2017) and human diabetics (Roshanravan *et al*, 2017) pointed toward the potential role of SCFAs to attenuate high blood pressure as well as chronic injury of the heart and kidneys. However, these suggested microbiome‐driven effects on blood pressure (Chen *et al*, 2020; Cook & Chappell, 2021) may also be indirectly driven by altered weight and glucose metabolism.

### Systemic nonmetabolic effects

In addition to the above systemic metabolite‐driven effects on host metabolism, microbial‐modulated metabolites may modify a variety of other physiological and pathological processes in numerous cells and organs. For example, in the brain, a mouse model of amyotrophic lateral sclerosis (ALS) featured dysbiosis in the gut preceding the motor symptoms (Blacher *et al*, 2019). A decrease of *A. muciniphila* in ALS‐bearing mice was accompanied by decreased systemic levels of nicotinamide, a metabolic disturbance that was also found in a cohort of ALS patients. *Akkermansia* or nicotinamide supplementation improved motor symptoms and triggered protective transcriptional neural pathways in the mouse ALS model, encouraging a future testing of this and other differentially abundant metabolites in human ALS clinical trials. Likewise, microbiome‐associated metabolites may modify disease course in other central nervous system disorders. The aggregation of the amyloid protein alpha‐synuclein (aSyn) is associated with several neurodegenerative disorders, including Parkinson's disease (PD). An amyloidogenic product secreted by *Enterobactericae* named “Curli” may increase the formation of aSyn‐aggregates, while colonizing an aSyn‐overexpressing mouse line with a Curli producing *E. coli* strain worsened the PD‐like motor and intestinal impairments (Sampson *et al*, 2020). Other metabolite alterations correlated with autism spectrum disorder (ASD). 5‐aminovaleric acid and taurine alleviated symptoms in an ASD mouse model and were suggested as candidates to modify behavioral patterns in ASD patients (Sharon *et al*, 2019). Altered bacterial tetrahydrobiopterin (BH4) synthesis was associated with behavioral disorders in a mouse ASD model, while targeted replenishment with BH4 improved ASD‐like behavioral deficits (Buffington *et al*, 2021). These interesting observations merit further mechanistic validation in mice and humans. In the kidneys, the tryptophan derivate indoxylsulfate was found to accumulate in subjects suffering from chronic kidney disease (CKD), thereby contributing to uremic symptoms (Devlin *et al*, 2016). A probiotic intervention with a *Lactobacillus* strain increased renal SCFA levels, resulting in protective effects from fibrosis and chronic renal dysfunction after ischemia‐reperfusion injury in mice. A subsequent administration of the probiotic to a cohort of CKD patients in a placebo‐controlled study reduced the decline of kidney function over the period of observation (Zhu *et al*, 2021). Other examples of systemic metabolite‐induced effects are reviewed elsewhere (Needham *et al*, 2020; Rossi *et al*, 2020; Schupack *et al*, 2022).

## Microbial metabolites shaping host immunity

**Figure 3:**
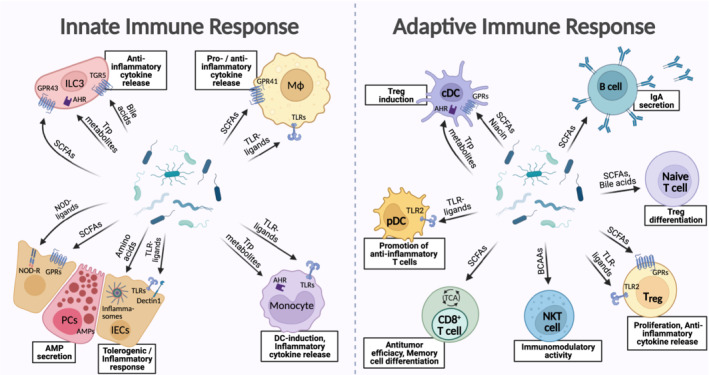
Microbiome‐modulated metabolites modify the innate and adaptive immune response Metabolite signaling contributes to a balance between tolerogenic and inflammatory immune reactions. Intestinal epithelial cells (IECs) sense short chain fatty acids (SCFAs); and other microbial metabolites through toll‐like receptors (TLRs), nucleotide binding and oligomerization domain receptors (NLRs), inflammasomes and Dectin1, in inducing tolerance to commensals versus inflammatory responses against pathogens. The secretion of antimicrobial peptides (AMPs) from Paneth cells and IECs upon sensing of amino acids, SCFAs, and microbial‐associated molecular patterns by TLRs‐ and NLRs contributes to protecting the gut from pathobiont bloom and invasion. Both monocytes and macrophages can recognize SCFAs, tryptophan metabolites, or TLR‐ligands affecting the differentiation of surrounding dendritic cells (DCs) or triggering the release of either pro‐ or anti‐inflammatory cytokines. In type 3 innate lymphoid cells 3 (ILC3s), recognition of SCFAs, bile acids, and aryl hydrocarbon receptor (AHR)‐ligands through specialized cell surface receptors also leads to the promotion of anti‐inflammatory cytokine patterns. Adaptive immune cells also interact with microbial metabolites. Classical dendritic cells (cDCs) recognize components of Gram‐positive bacteria through TLR2, while plasmacytoid dendritic cells (pDCs) express AHR and G‐protein coupled receptors (GPRs) interacting with tryptophan metabolites, SCFAs, and niacin. These antigen‐presenting cell (APC)‐signals modulate the activation of B and T cells. T cells also directly react to microbial signals in shaping their immunomodulatory phenotype: the differentiation of naïve T cells to regulatory T cells (Tregs) is reinforced by SCFAs and bile acids. Also, Tregs sense SCFAs and bacterial components through GPRs and TLR2 resulting in proliferation and the secretion of anti‐inflammatory cytokines. SCFAs can lead to an immunoglobulin A (IgA)‐class switch in B cells, so that secreted IgA in the intestinal lumen can counteract bacterial dissemination. Natural killer T (NKT) cells are polarized toward an immunomodulatory state counteracting inflammation in reaction to branched‐chain amino acids (BCAAs). CD8^+^ T cell sensing of SCFAs regulates their differentiation into memory cells and cytotoxic anti‐tumor activity. AHR, aryl hydrocarbon receptor; AMP, antimicrobial peptide; BCAA, branched‐chain amino acid; cDC, classical dendritic cell; DC, dendritic cell; GPR, G‐protein coupled receptor; IEC, intestinal epithelial cell; IgA, immunoglobulin A; MΦ, macrophage; NKT, natural killer T cell; NLR, nucleotide binding and oligomerization domain receptor; NOD, nucleotide binding and oligomerization domain; PC, Paneth cell; pDC, plasmacytoid dendritic cell; SCFA, short chain fatty acid; TCA, tricarboxylic acid cycle; TGR5, G protein‐coupled bile acid receptor 1; TLR, toll‐like receptor; TMAO, trimethylamine‐oxide; Treg, regulatory T cell; Trp, tryptophan. (Created with BioRender.com).

Metabolite impacts on the mammalian immune system can be both local and systemic and are central in shaping microbiome‐host interactions and their physiological consequences. A variety of microbial recognition patterns of the host's innate and adaptive immune mechanisms are able to induce a myriad of host responses ranging from anti‐microbial activity to maintenance of tolerance and commensalism (Fig 3). While microbial surface components and nucleic acids are central in innate immune system microbial recognition, their metabolites constitute important additional means of signaling with and modulation of both innate and adaptive immune cells.

### Modulation of the innate immune system

The innate immune system is tasked with sensing of microbiome commensals, helping the host to differentiate between “friend and foe” signals, and directing responses to these signals that can range from tolerance to inflammation. Microbial‐modulated metabolites are involved in these processes. IECs, increasingly considered an integral part of the mucosal immune system, serve as a critical communication hub, together with professional immune cells, in sensing and reacting to commensals and pathogens (Pott & Hornef, 2012; Soderholm & Pedicord, 2019). The most common specialized luminal receptors for the interaction with molecules of microbial origin are the G‐protein coupled receptors (GPRs) 41, 43, and 109 sensing SCFAs, as well as TLRs recognizing a variety of bacterial surface structures (Zheng *et al*, 2022). Several other surface receptors such as Dectin‐1 (Gantner *et al*, 2003), CD14 (Jiang *et al*, 2005), and CD36 (Hoebe *et al*, 2005) closely interact with TLRs in sensing components of putative pathogens. Additionally, intracellular detection and signaling is mediated by NOD‐like receptors (NLRs) and multiprotein‐complexes involving certain NLRs, termed inflammasomes. Collectively, integration of these diverse signals leads to tolerogenic versus inflammatory reactions (Rakoff‐Nahoum *et al*, 2004; Macho Fernandez *et al*, 2011; Macia *et al*, 2015; Wang *et al*, 2020).

IEC crosstalk with bacterial metabolites is also essential for the secretion of AMPs. IEC‐sensing of SCFAs (Zhao *et al*, 2018; Hayashi *et al*, 2021a) or the amino acid taurine activating NRLP6‐inflammasomes (Levy *et al*, 2015) trigger the release of AMPs. This increases the resistance toward noxious toxin‐producing *C. difficile* strains (Hayashi *et al*, 2021a) and attenuates the severity of experimentally induced colitis (Levy *et al*, 2015) in mice. Paneth cells can also independently respond with AMP‐secretion upon microbial components sensed through TLRs on their surface or intracellularly through the NOD2 pattern recognition receptor (Ogura *et al*, 2003). This contributes to lower intestinal penetration of bacteria and decreases colonization with pathobionts (Vaishnava *et al*, 2008).

However, the recognition of bacterial components through TLRs on host cells can have detrimental outcomes in cases of chronic inflammation, such as ulcerative colitis (UC). As such, TLR4 is upregulated in colon tumors found in UC‐patients as well as in a mouse model of inflammation‐associated colon cancer (AOM‐DSS; Fukata *et al*, 2007). The latter provides a potential mechanism linking TLR4 activation to the COX‐PGE‐EGFR pathway in promoting cancer (Fukata *et al*, 2007).

The innate immune system is also involved in responses to molecules of bacterial origin at sites remote from their production. Myeloid cells have emerged as critical hubs sensing and responding to bacterial signals. Innate lymphoid cells (ILCs) can be found in the subepithelial layers of organs harboring microbial communities. Their interaction with a variety of microbial products modulates their impact on innate immune response mechanisms. For example, SCFAs can influence type 3 innate lymphoid cells (ILC3s) through histone‐deacetylase (HDAC)‐inhibition (Yang *et al*, 2020), GPR43‐binding (Chun *et al*, 2019) or triggering of neutrophils to induce inflammasome activation (Fachi *et al*, 2020). In mouse models, this resulted in a protective effect from gut inflammatory injury (Chun *et al*, 2019; Yang *et al*, 2020), and infection (Chun *et al*, 2019; Fachi *et al*, 2020). Moreover, ILC3s exert anti‐inflammatory effects upon interaction with primary bile acids through TGR5 (Qi *et al*, 2019) as well as bacterial tryptophan derivatives binding AHR (Laurans *et al*, 2018; Hendrikx *et al*, 2019).

The liver serves as an immunologic “first‐pass” gate keeper, by responding to an array of metabolites influxing from the gut through the portal vein. Kupffer cells, the tissue‐resident macrophages in the liver, can respond to bacterial products while inducing distinct responses in their surrounding cell populations. Kupffer cell sensing of bacterial components through TLRs plays a role in triggering liver inflammation during liver steatosis (Henao‐Mejia *et al*, 2012; Carpino *et al*, 2020) as well as in acute liver failure (Kolodziejczyk *et al*, 2020). On the other hand, the abrogation of bacterial signaling through TLR‐ligands in germ‐free animals may lead to an increased susceptibility to hepatic fibrosis (Mazagova *et al*, 2015).

Adipose tissue (AT) also contains macrophages exerting an important role in regulating local inflammation and lipid storage. Their inflammatory responses can be modified by certain molecules of microbial origin. SCFAs may cause AT‐macrophages to abolish the secretion of various pro‐inflammatory cytokines and chemokines (Al‐Lahham *et al*, 2012; Ohira *et al*, 2013), including the adipokine resistin (Curat *et al*, 2006; Al‐Lahham *et al*, 2010). Increased gut permeability during obesity is driven by dietary compounds, perturbations of gut microbial communities, and the local immune response of the host, leading to endotoxemia (Serino *et al*, 2012; Tilg *et al*, 2020). In the AT, this results in a TLR4‐dependent recruitment of macrophages, triggering inflammation (Caesar *et al*, 2015). In addition, sensing of microbial patterns through TLR4 as well as NRLP3 in AT may trigger mitochondrial dysfunction upregulating fat storage processes, closely related to metabolic syndrome (Okla *et al*, 2018).

### Modulation of the adaptive immune system

The adaptive immune response is an antigen‐specific and memory‐inducing immune reaction that evolved in vertebrates. Co‐evolution with complex microbial communities contributes to proper development and function of the adaptive immune response (McFall‐Ngai, 2007), while defective microbial colonization leads to an altered adaptive immunity, thereby triggering susceptibility toward infections (Mazmanian *et al*, 2005; Hall *et al*, 2008; Ivanov *et al*, 2009) and reduced efficiency of vaccinations (Korpe & Petri, 2012). Therefore, it is not surprising that microbial metabolites may shape functions of the adaptive immune system, in particular those involving several T cell subsets.

For example, SCFAs may impact intestinal T cell function by inducing their differentiation into anti‐inflammatory regulatory T cells (Tregs), while preventing a shift toward the pro‐inflammatory Th1/17 phenotype. This effect is mediated through HDAC‐inhibition (Arpaia *et al*, 2013) and metabolic reprogramming toward fatty acid oxidation (Hao *et al*, 2021). Similarly, SCFAs signaling via GPRs may directly target intestinal Tregs, leading to their increase in number and proliferative capacity through HDAC‐inhibition (Smith *et al*, 2013). To harness the local immunoregulatory potential of SCFAs, attempts of a postbiotic treatment were conducted in inflammatory bowel disease involving local administration of butyrate to patients suffering from ulcerous proctosigmoiditis (Scheppach *et al*, 1992; Scheppach & German‐Austrian SCFA Study Group, 1996). However, only a few of the heterogeneous and rather small‐scale studies were able to detect a decrease of inflammatory activity (Jamka *et al*, 2021).

The potential of SCFAs to facilitate an extrathymic development of anti‐inflammatory Tregs (Arpaia *et al*, 2013) can promote delivering immuno‐metabolic signals from gut microbes to distant effector organs. In mouse AT, butyrate leads to a Treg‐triggered alleviation of steato‐inflammation (Sato *et al*, 2020). In high‐fat diet induced obesity, SCFA‐triggered Tregs elicit weight loss and improve insulin sensitivity (Mandaliya *et al*, 2021). Inflammation in the course of pulmonary allergic hypersensitivity modeled in mice responds to increased SCFA levels leading to induction of higher numbers of Tregs (Trompette *et al*, 2014; Zaiss *et al*, 2015). In the pancreas, Tregs may alleviate the destruction of insulin‐producing β‐cells observed in the NOD1 mouse model of type‐1 Diabetes mellitus (T1DM). This effect is dependent on gut microbial SCFAs and AHR‐ligands, which, in turn induce pancreatic islet secretion of antimicrobial peptides (AMPs; Sun *et al*, 2015; Miani *et al*, 2018).

Importantly, SCFAs may also contribute to other adaptive immune effects. SCFAs may induce the inflammatory Th1/Th17 CD4 T cell phenotype in the setting of *C. rodentium* infection (Kim *et al*, 2013). Also, they may promote urethritis (Park *et al*, 2016), stimulate cytotoxic CD8^+^ lymphocytes (Luu *et al*, 2018), possibly contributing to their anti‐tumor effects, and impact B cell‐ and antibody‐related responses. An activation of GPR41 and GPR109 in DCs stimulates B cells to an IgA‐class switch and secretion of IgA into the gut lumen, thereby inhibiting bacterial dissemination into distant organs in the course of experimental colitis (Isobe *et al*, 2020).

Bile acids modified by gut commensals may also impact the differentiation and activity of T cells. Iso‐desoxycholic acid induces anti‐inflammatory FoxP3^+^/RoRγt^+^ Tregs by blocking nuclear FXR‐signaling in interacting DCs (Campbell *et al*, 2020). Both 3‐oxo lithocholic acid (LCA) and iso‐allo‐LCA direct T cells toward an anti‐inflammatory Treg phenotype while preventing Th17 differentiation (Hang *et al*, 2019). The mechanism by which both bile acids induce Tregs includes enhancement of mitochondrial function, resulting in transcription through histone acetylation at the promoter of FoxP3 (Hang *et al*, 2019).

While sensing of bacterial components by TLRs is mainly attributed to innate immune functions, it can also have an impact on inflammatory pathways driven by adaptive immune responses. Polysaccharide A (PSA), a component of the capsule of *B. fragilis*, induces a tolerogenic Treg phenotype through multiple mechanisms, including binding to TLR2 on plasmacytoid dendritic cells (pDCs; Dasgupta *et al*, 2014) or FoxP3^+^ CD4^+^ T cells (Round *et al*, 2011), as well as directly promoting the secretion of the anti‐inflammatory cytokine Il‐10 from T cells (Mazmanian *et al*, 2008). In the pancreas, microbial components interacting with different TLRs in mouse models can either exacerbate or alleviate autoimmune injury, a hallmark of T1DM. TLR2 may induce a microbiome‐dependent inflammatory impairment, while TLR4 exerts a protective tolerogenic effect on pancreatic islets (Burrows *et al*, 2015).

Niacin, produced by commensal bacteria in the gut from nicotinamide adenine dinucleotide (NAD), can activate local DCs and macrophages by interacting with their membrane receptor GPR109a (Singh *et al*, 2014). This subsequently drives a differentiation of naïve T cells into Tregs (Singh *et al*, 2014). These effects reduce gut inflammation and the subsequent carcinogenesis induced in the azoxymethane‐dextran sodium sulfate (AOM‐DSS) mouse model (Singh *et al*, 2014). Bacterial tryptophan metabolites signaling through AHR in the gut can induce CD4^+^8^+^ double‐positive intraepithelial lymphocytes (IEL; Cervantes‐Barragan *et al*, 2017) as well as non‐monocyte‐derived DCs (Kinnebrew *et al*, 2012), both associated with reduced intestinal inflammation. In the skin epithelia, supplementation with the tryptophan derivate indole‐3‐aldehyde (IAId) results in reduced inflammation caused by a mouse model of atopic dermatitis (Yu *et al*, 2019). This bacterial metabolite is decreased in the skin of people suffering from this disease (Yu *et al*, 2019).

Other metabolites of bacterial origin induce effector T cells. Glycosphingolipids, generated from BCAAs, lead to the development of NKT‐cells in the gut toward an immunomodulatory state capable of ameliorating colitis in mice (Oh *et al*, 2021). SCFA derivatives can be incorporated into the TCA of CD8^+^ T cells to enhance their metabolic capacity through mitochondrial oxidative phosphorylation, eventually improving survival and leading to polarization toward memory cells upon activation through antigen encounter (Bachem *et al*, 2019). Harnessing this beneficial effect may enable to sustain T cell memory after vaccinations or enable *in vitro* induction of memory chimeric antigen receptor (CAR) T cells in optimizing cancer immunotherapy.

## Challenges and perspectives in the study of microbiome‐associated metabolites

As outlined in this review, metabolites derived from or modulated by microbial commensals impact their eukaryote host in a variety of local and systemic manners, which may affect host immunity, metabolism, and disease susceptibility. Disentangling the underlying mechanisms of these complex metabolite effects and harnessing them as microbiome‐targeted interventions remains complicated and elusive in most cases (Box 1).

Mechanistically studying metabolite impacts on host physiology and disease is complicated by multiple challenges. These include, among others, the lack of standardization of analytical pipelines, mainly those involving microbiome sequencing (Beresford‐Jones *et al*, 2022), and difficulties in culturing of many commensals, resulting in over‐reliance on their genomic characterization without sufficient evidence of causality (Cani, 2018). Moreover, functional analyses of bacterial metabolism, usually performed within the *in vitro* setting, does not account for metabolite impacts on the host and on neighboring microbes. Additionally, characterizing the “rules of engagement” of complex networks of microbial communities and their broad spectrum of metabolite secretomes necessitates the development of sophisticated computational tools and machine learning processes predicting the impact of metabolite consortia on distinct physiological responses.

Many approaches striving to determine metabolite causation and molecular mechanisms impacting disease processes heavily rely on rodent models. However, translational generalization of their findings to the human setting is impaired by the limited shared bacterial taxa (Chung *et al*, 2012), difficulties in reproducibility attributed to variations in microbial communities throughout animal vending and housing institutions (Beresford‐Jones *et al*, 2022), constrains in colonization and function upon transfer of human bacteria into mice (Lundberg *et al*, 2020) and variable effects of microbial metabolites on host physiology across species (Koh & Bäckhed, 2020). Furthermore, in some cases, secretion of microbial metabolites and their downstream impact on the host are investigated using unphysiological doses and application routes, thereby reducing the potential to translate findings from animal studies into the human setting. In human studies, a major challenge includes a high inter‐individual variability in microbiome composition and metabolite landscape, stemming from a multitude of environmental, immune, and genomic variations such as diet, ethnicity, and geography.

Regardless of these difficulties and challenges, the insight into the ubiquitous functions of microbially modulated metabolites has opened a new window of opportunity toward improving human health. In contrast to attempts to define a “core microbiome” by taxonomic similarity (Neu *et al*, 2021), the shared metabolite landscape of microbes and their host may allow a better functional classification of microbiome contributions in distinct clinical contexts (Beresford‐Jones *et al*, 2022). Metabolites derived from or modified by commensals may play central roles in driving “personalized” interventions. Within the Personalized Nutrition Project, microbial features were harnessed to develop a prediction platform of glycemic responses (Zeevi *et al*, 2015). This outperformed common dietary approaches in an intervention targeting the postprandial glucose levels in healthy and (pre‐)diabetic subjects (Zeevi *et al*, 2015; Ben‐Yacov *et al*, 2021; Rein *et al*, 2022). A diet leading to the blossom of strains with the capacity of carbohydrate fermentation (Zeevi *et al*, 2015) as well as propionate production (Rein *et al*, 2022) was associated with metabolic improvements, corresponding to previous reports (Qin *et al*, 2012; Louis & Flint, 2017). However, a dietary pattern promoting strains from the *Alistipes* phylum and *Bacteriodetes* genus was found to have detrimental metabolic effects, contradicting prior studies which associated these microbial signatures with leanness (Turnbaugh *et al*, 2006, 2009). This underlines that personalized approaches focused on the interplay of diet and microbiome have the potential to guide future strategies in the prevention and treatment of metabolic disorders. Moreover, probiotic supplementation steadily increases in popularity, despite unequivocal results from studies with methodological quality issues, evidence of colonization resistance, and a lack of assessment of long‐term adverse outcomes (Suez *et al*, 2019). Compounds secreted by probiotics play an important role in inhibiting the reconstitution of the gut microbiome after antibiotic therapy in susceptible subjects (Suez *et al*, 2018). The inter‐individual variability in effects of probiotics adds a perspective of a personalized approach in refining their use, which is currently based on inconclusive evidence (McFarland, 2014). Eventually, postbiotic interventions can be tailored to balance disturbances in the metabolic function of the resident microbiome. A selective supplementation of metabolites, as demonstrated in preclinical models of weight gain after dieting and smoking cessation (Thaiss *et al*, 2016a; Fluhr *et al*, 2021), is expected to be increasingly researched also in the human setting, with an outlook to be incorporated into the precision medicine toolbox.

## Author contributions

**Eran Elinav:** Conceptualization; supervision; funding acquisition; writing – original draft; writing – review and editing. **Igor Spivak:** Conceptualization; data curation; writing – original draft; writing – review and editing. **Leviel Fluhr:** Conceptualization; data curation; writing – original draft; writing – review and editing.

## Disclosure and competing interests statement

EE is a scientific founder of DayTwo and BiomX, and a paid consultant to Hello Inside and Aposense. The remaining authors declare no competing interests.

## References

[embr202255664-bib-0001] Agostini L , Down PF , Murison J , Wrong OM (1972) Faecal ammonia and pH during lactulose administration in man: comparison with other cathartics. Gut 13: 859–866 4646289 10.1136/gut.13.11.859 PMC1412409

[embr202255664-bib-0002] Aguilar‐Toalá JE , Garcia‐Varela R , Garcia HS , Mata‐Haro V , González‐Córdova AF , Vallejo‐Cordoba B , Hernández‐Mendoza A (2018) Postbiotics: an evolving term within the functional foods field. Trends Food Sci Technol 75: 105–114

[embr202255664-bib-0003] Aguilar‐Toalá JE , Arioli S , Behare P , Belzer C , Berni Canani R , Chatel J‐M , D'Auria E , de Freitas MQ , Elinav E , Esmerino EA *et al* (2021) Postbiotics – when simplification fails to clarify. Nat Rev Gastroenterol Hepatol 18: 825–826 34556825 10.1038/s41575-021-00521-6

[embr202255664-bib-0004] Agus A , Planchais J , Sokol H (2018) Gut microbiota regulation of tryptophan metabolism in health and disease. Cell Host Microbe 23: 716–724 29902437 10.1016/j.chom.2018.05.003

[embr202255664-bib-0005] Agus A , Clément K , Sokol H (2021) Gut microbiota‐derived metabolites as central regulators in metabolic disorders. Gut 70: 1174–1182 33272977 10.1136/gutjnl-2020-323071 PMC8108286

[embr202255664-bib-0006] Alberti KGMM , Zimmet P , Shaw J (2006) Metabolic syndrome–a new world‐wide definition. A Consensus Statement from the International Diabetes Federation. Diabet Med 23: 469–480 16681555 10.1111/j.1464-5491.2006.01858.x

[embr202255664-bib-0007] Aldunate M , Tyssen D , Johnson A , Zakir T , Sonza S , Moench T , Cone R , Tachedjian G (2013) Vaginal concentrations of lactic acid potently inactivate HIV. J Antimicrob Chemother 68: 2015–2025 23657804 10.1093/jac/dkt156 PMC3743514

[embr202255664-bib-0008] Alexander M , Turnbaugh PJ (2020) Deconstructing mechanisms of diet‐microbiome‐immune interactions. Immunity 53: 264–276 32814025 10.1016/j.immuni.2020.07.015 PMC7441819

[embr202255664-bib-0009] Al‐Lahham SH , Roelofsen H , Priebe M , Weening D , Dijkstra M , Hoek A , Rezaee F , Venema K , Vonk RJ (2010) Regulation of adipokine production in human adipose tissue by propionic acid. Eur J Clin Invest 40: 401–407 20353437 10.1111/j.1365-2362.2010.02278.x

[embr202255664-bib-0010] Al‐Lahham S , Roelofsen H , Rezaee F , Weening D , Hoek A , Vonk R , Venema K (2012) Propionic acid affects immune status and metabolism in adipose tissue from overweight subjects. Eur J Clin Invest 42: 357–364 21913915 10.1111/j.1365-2362.2011.02590.x

[embr202255664-bib-0011] Andonegui G , Kerfoot SM , McNagny K , Ebbert KVJ , Patel KD , Kubes P (2005) Platelets express functional Toll‐like receptor‐4. Blood 106: 2417–2423 15961512 10.1182/blood-2005-03-0916

[embr202255664-bib-0012] Apostolopoulou M , Gordillo R , Koliaki C , Gancheva S , Jelenik T , De Filippo E , Herder C , Markgraf D , Jankowiak F , Esposito I *et al* (2018) Specific hepatic sphingolipids relate to insulin resistance, oxidative stress, and inflammation in nonalcoholic steatohepatitis. Diabetes Care 41: 1235–1243 29602794 10.2337/dc17-1318

[embr202255664-bib-0013] Araújo JR , Tazi A , Burlen‐Defranoux O , Vichier‐Guerre S , Nigro G , Licandro H , Demignot S , Sansonetti PJ (2020) Fermentation products of commensal bacteria alter enterocyte lipid metabolism. Cell Host Microbe 27: 358–375 32101704 10.1016/j.chom.2020.01.028

[embr202255664-bib-0014] Arpaia N , Campbell C , Fan X , Dikiy S , van der Veeken J , deRoos P , Liu H , Cross JR , Pfeffer K , Coffer PJ *et al* (2013) Metabolites produced by commensal bacteria promote peripheral regulatory T‐cell generation. Nature 504: 451–455 24226773 10.1038/nature12726 PMC3869884

[embr202255664-bib-0015] Aslam R , Speck ER , Kim M , Crow AR , Bang KWA , Nestel FP , Ni H , Lazarus AH , Freedman J , Semple JW (2006) Platelet Toll‐like receptor expression modulates lipopolysaccharide‐induced thrombocytopenia and tumor necrosis factor‐alpha production *in vivo* . Blood 107: 637–641 16179373 10.1182/blood-2005-06-2202

[embr202255664-bib-0016] Bachem A , Makhlouf C , Binger KJ , de Souza DP , Tull D , Hochheiser K , Whitney PG , Fernandez‐Ruiz D , Dähling S , Kastenmüller W *et al* (2019) Microbiota‐derived short‐chain fatty acids promote the memory potential of antigen‐activated CD8^+^ T cells. Immunity 51: 285–297 31272808 10.1016/j.immuni.2019.06.002

[embr202255664-bib-0017] Bajaj JS (2019) Alcohol, liver disease and the gut microbiota. Nat Rev Gastroenterol Hepatol 16: 235–246 30643227 10.1038/s41575-018-0099-1

[embr202255664-bib-0018] Ben‐Yacov O , Godneva A , Rein M , Shilo S , Kolobkov D , Koren N , Cohen Dolev N , Travinsky Shmul T , Wolf BC , Kosower N *et al* (2021) Personalized postprandial glucose response‐targeting diet versus mediterranean diet for glycemic control in prediabetes. Diabetes Care 44: 1980–1991 34301736 10.2337/dc21-0162

[embr202255664-bib-0019] Beresford‐Jones BS , Forster SC , Stares MD , Notley G , Viciani E , Browne HP , Boehmler DJ , Soderholm AT , Kumar N , Vervier K *et al* (2022) The mouse gastrointestinal bacteria catalogue enables translation between the mouse and human gut microbiotas via functional mapping. Cell Host Microbe 30: 124–138 34971560 10.1016/j.chom.2021.12.003 PMC8763404

[embr202255664-bib-0020] Bergman EN (1990) Energy contributions of volatile fatty acids from the gastrointestinal tract in various species. Physiol Rev 70: 567–590 2181501 10.1152/physrev.1990.70.2.567

[embr202255664-bib-0021] den Besten G , van Eunen K , Groen AK , Venema K , Reijngoud D‐J , Bakker BM (2013a) The role of short‐chain fatty acids in the interplay between diet, gut microbiota, and host energy metabolism. J Lipid Res 54: 2325–2340 23821742 10.1194/jlr.R036012 PMC3735932

[embr202255664-bib-0022] den Besten G , Lange K , Havinga R , van Dijk TH , Gerding A , van Eunen K , Müller M , Groen AK , Hooiveld GJ , Bakker BM *et al* (2013b) Gut‐derived short‐chain fatty acids are vividly assimilated into host carbohydrates and lipids. Am J Physiol Gastrointest Liver Physiol 305: G900‐10 24136789 10.1152/ajpgi.00265.2013

[embr202255664-bib-0023] den Besten G , Bleeker A , Gerding A , van Eunen K , Havinga R , van Dijk TH , Oosterveer MH , Jonker JW , Groen AK , Reijngoud D‐J *et al* (2015) Short‐chain fatty acids protect against high‐fat diet‐induced obesity via a PPARγ‐dependent switch from lipogenesis to fat oxidation. Diabetes 64: 2398–2408 25695945 10.2337/db14-1213

[embr202255664-bib-0024] Beyaz S , Chung C , Mou H , Bauer‐Rowe KE , Xifaras ME , Ergin I , Dohnalova L , Biton M , Shekhar K , Eskiocak O *et al* (2021) Dietary suppression of MHC class II expression in intestinal epithelial cells enhances intestinal tumorigenesis. Cell Stem Cell 28: 1922–1935 34529935 10.1016/j.stem.2021.08.007 PMC8650761

[embr202255664-bib-0025] Bhattarai Y , Williams BB , Battaglioli EJ , Whitaker WR , Till L , Grover M , Linden DR , Akiba Y , Kandimalla KK , Zachos NC *et al* (2018) Gut microbiota‐produced tryptamine activates an epithelial G‐protein‐coupled receptor to increase colonic secretion. Cell Host Microbe 23: 775–785 29902441 10.1016/j.chom.2018.05.004 PMC6055526

[embr202255664-bib-0026] Biesalski HK (2016) Nutrition meets the microbiome: micronutrients and the microbiota. Ann N Y Acad Sci 1372: 53–64 27362360 10.1111/nyas.13145

[embr202255664-bib-0027] Birchenough GMH , Nyström EEL , Johansson MEV , Hansson GC (2016) A sentinel goblet cell guards the colonic crypt by triggering Nlrp6‐dependent Muc2 secretion. Science 352: 1535–1542 27339979 10.1126/science.aaf7419 PMC5148821

[embr202255664-bib-0028] Björntorp P , Hood B (1966) Studies on adipose tissue from obese patients with or without diabetes mellitus. I. Release of glycerol and free fatty acids. Acta Med Scand 179: 221–227 5908977 10.1111/j.0954-6820.1966.tb05451.x

[embr202255664-bib-0029] Blacher E , Bashiardes S , Shapiro H , Rothschild D , Mor U , Dori‐Bachash M , Kleimeyer C , Moresi C , Harnik Y , Zur M *et al* (2019) Potential roles of gut microbiome and metabolites in modulating ALS in mice. Nature 572: 474–480 31330533 10.1038/s41586-019-1443-5

[embr202255664-bib-0030] Borghi M , Puccetti M , Pariano M , Renga G , Stincardini C , Ricci M , Giovagnoli S , Costantini C , Romani L (2020) Tryptophan as a central hub for host/microbial symbiosis. Int J Tryptophan Res 13: 1178646920919755 32435131 10.1177/1178646920919755 PMC7225782

[embr202255664-bib-0031] Broeders EPM , Nascimento EBM , Havekes B , Brans B , Roumans KHM , Tailleux A , Schaart G , Kouach M , Charton J , Deprez B *et al* (2015) The bile acid chenodeoxycholic acid increases human brown adipose tissue activity. Cell Metab 22: 418–426 26235421 10.1016/j.cmet.2015.07.002

[embr202255664-bib-0032] Buffie CG , Bucci V , Stein RR , McKenney PT , Ling L , Gobourne A , No D , Liu H , Kinnebrew M , Viale A *et al* (2015) Precision microbiome reconstitution restores bile acid mediated resistance to *Clostridium difficile* . Nature 517: 205–208 25337874 10.1038/nature13828 PMC4354891

[embr202255664-bib-0033] Buffington SA , Dooling SW , Sgritta M , Noecker C , Murillo OD , Felice DF , Turnbaugh PJ , Costa‐Mattioli M (2021) Dissecting the contribution of host genetics and the microbiome in complex behaviors. Cell 184: 1740–1756 33705688 10.1016/j.cell.2021.02.009 PMC8996745

[embr202255664-bib-0034] Bugaut M (1987) Occurrence, absorption and metabolism of short chain fatty acids in the digestive tract of mammals. Comp Biochem Physiol B 86: 439–472 3297476 10.1016/0305-0491(87)90433-0

[embr202255664-bib-0035] Burrows MP , Volchkov P , Kobayashi KS , Chervonsky AV (2015) Microbiota regulates type 1 diabetes through Toll‐like receptors. Proc Natl Acad Sci U S A 112: 9973–9977 26216961 10.1073/pnas.1508740112 PMC4538618

[embr202255664-bib-0036] Caesar R , Tremaroli V , Kovatcheva‐Datchary P , Cani PD , Bäckhed F (2015) Crosstalk between gut microbiota and dietary lipids aggravates WAT inflammation through TLR signaling. Cell Metab 22: 658–668 26321659 10.1016/j.cmet.2015.07.026 PMC4598654

[embr202255664-bib-0037] Campbell C , McKenney PT , Konstantinovsky D , Isaeva OI , Schizas M , Verter J , Mai C , Jin W‐B , Guo C‐J , Violante S *et al* (2020) Bacterial metabolism of bile acids promotes generation of peripheral regulatory T cells. Nature 581: 475–479 32461639 10.1038/s41586-020-2193-0 PMC7540721

[embr202255664-bib-0038] Cani PD (2018) Human gut microbiome: hopes, threats and promises. Gut 67: 1716–1725 29934437 10.1136/gutjnl-2018-316723 PMC6109275

[embr202255664-bib-0039] Cani PD , Van Hul M , Lefort C , Depommier C , Rastelli M , Everard A (2019) Microbial regulation of organismal energy homeostasis. Nat Metab 1: 34–46 32694818 10.1038/s42255-018-0017-4

[embr202255664-bib-0040] Carpino G , Del Ben M , Pastori D , Carnevale R , Baratta F , Overi D , Francis H , Cardinale V , Onori P , Safarikia S *et al* (2020) Increased liver localization of lipopolysaccharides in human and experimental NAFLD. Hepatology 72: 470–485 31808577 10.1002/hep.31056

[embr202255664-bib-0041] Carrasco‐Pozo C , Morales P , Gotteland M (2013) Polyphenols protect the epithelial barrier function of Caco‐2 cells exposed to indomethacin through the modulation of occludin and zonula occludens‐1 expression. J Agric Food Chem 61: 5291–5297 23668856 10.1021/jf400150p

[embr202255664-bib-0042] Cervantes‐Barragan L , Chai JN , Tianero MD , Di Luccia B , Ahern PP , Merriman J , Cortez VS , Caparon MG , Donia MS , Gilfillan S *et al* (2017) Lactobacillus reuteri induces gut intraepithelial CD4^+^CD8αα^+^ T cells. Science 357: 806–810 28775213 10.1126/science.aah5825 PMC5687812

[embr202255664-bib-0043] Chaluvadi S , Hotchkiss AT , Yam KL (2016) Chapter 36 – Gut microbiota: impact of probiotics, prebiotics, synbiotics, pharmabiotics, and postbiotics on human health. In Probiotics, Prebiotics, and Synbiotics, RR Watson , VR Preedy (eds), pp 515–523. London: Academic Press

[embr202255664-bib-0044] Chambers ES , Byrne CS , Rugyendo A , Morrison DJ , Preston T , Tedford C , Bell JD , Thomas L , Akbar AN , Riddell NE *et al* (2019) The effects of dietary supplementation with inulin and inulin‐propionate ester on hepatic steatosis in adults with non‐alcoholic fatty liver disease. Diabetes Obes Metab 21: 372–376 30098126 10.1111/dom.13500 PMC6667894

[embr202255664-bib-0045] Chen R , Xu Y , Wu P , Zhou H , Lasanajak Y , Fang Y , Tang L , Ye L , Li X , Cai Z *et al* (2019a) Transplantation of fecal microbiota rich in short chain fatty acids and butyric acid treat cerebral ischemic stroke by regulating gut microbiota. Pharmacol Res 148: 104403 31425750 10.1016/j.phrs.2019.104403

[embr202255664-bib-0046] Chen S , Henderson A , Petriello MC , Romano KA , Gearing M , Miao J , Schell M , Sandoval‐Espinola WJ , Tao J , Sha B *et al* (2019b) Trimethylamine N‐oxide binds and activates PERK to promote metabolic dysfunction. Cell Metab 30: 1141–1151 31543404 10.1016/j.cmet.2019.08.021

[embr202255664-bib-0047] Chen X , Li P , Liu M , Zheng H , He Y , Chen M‐X , Tang W , Yue X , Huang Y , Zhuang L *et al* (2020) Gut dysbiosis induces the development of pre‐eclampsia through bacterial translocation. Gut 69: 513–522 31900289 10.1136/gutjnl-2019-319101

[embr202255664-bib-0048] Chen Y‐M , Liu Y , Zhou R‐F , Chen X‐L , Wang C , Tan X‐Y , Wang L‐J , Zheng R‐D , Zhang H‐W , Ling W‐H *et al* (2016) Associations of gut‐flora‐dependent metabolite trimethylamine‐N‐oxide, betaine and choline with non‐alcoholic fatty liver disease in adults. Sci Rep 6: 19076 26743949 10.1038/srep19076 PMC4705470

[embr202255664-bib-0049] Chhibber‐Goel J , Gaur A , Singhal V , Parakh N , Bhargava B , Sharma A (2016) The complex metabolism of trimethylamine in humans: endogenous and exogenous sources. Expert Rev Mol Med 18: e8 27876109 10.1017/erm.2016.19

[embr202255664-bib-0050] Chun E , Lavoie S , Fonseca‐Pereira D , Bae S , Michaud M , Hoveyda HR , Fraser GL , Gallini Comeau CA , Glickman JN , Fuller MH *et al* (2019) Metabolite‐sensing receptor Ffar2 regulates colonic group 3 innate lymphoid cells and gut immunity. Immunity 51: 871–884 31628054 10.1016/j.immuni.2019.09.014 PMC6901086

[embr202255664-bib-0051] Chung H , Pamp SJ , Hill JA , Surana NK , Edelman SM , Troy EB , Reading NC , Villablanca EJ , Wang S , Mora JR *et al* (2012) Gut immune maturation depends on colonization with a host‐specific microbiota. Cell 149: 1578–1593 22726443 10.1016/j.cell.2012.04.037 PMC3442780

[embr202255664-bib-0052] Conti C , Malacrino C & Mastromarino P (2009) Inhibition of herpes simplex virus type 2 by vaginal lactobacilli. J Physiol Pharmacol 60 Suppl 6: 19–26 20224147

[embr202255664-bib-0053] Cook KL , Chappell MC (2021) Gut dysbiosis and hypertension: is it cause or effect? J Hypertens 39: 1768–1770 34397625 10.1097/HJH.0000000000002908 PMC8371703

[embr202255664-bib-0054] Costello EK , Stagaman K , Dethlefsen L , Bohannan BJM , Relman DA (2012) The application of ecological theory toward an understanding of the human microbiome. Science 336: 1255–1262 22674335 10.1126/science.1224203 PMC4208626

[embr202255664-bib-0055] Coudray C , Bellanger J , Castiglia‐Delavaud C , Rémésy C , Vermorel M , Rayssignuier Y (1997) Effect of soluble or partly soluble dietary fibres supplementation on absorption and balance of calcium, magnesium, iron and zinc in healthy young men. Eur J Clin Nutr 51: 375–380 9192195 10.1038/sj.ejcn.1600417

[embr202255664-bib-0056] Cullin N , Azevedo Antunes C , Straussman R , Stein‐Thoeringer CK , Elinav E (2021) Microbiome and cancer. Cancer Cell 39: 1317–1341 34506740 10.1016/j.ccell.2021.08.006

[embr202255664-bib-0057] Curat CA , Wegner V , Sengenès C , Miranville A , Tonus C , Busse R , Bouloumié A (2006) Macrophages in human visceral adipose tissue: increased accumulation in obesity and a source of resistin and visfatin. Diabetologia 49: 744–747 16496121 10.1007/s00125-006-0173-z

[embr202255664-bib-0058] Dasgupta S , Erturk‐Hasdemir D , Ochoa‐Reparaz J , Reinecker H‐C , Kasper DL (2014) Plasmacytoid dendritic cells mediate anti‐inflammatory responses to a gut commensal molecule via both innate and adaptive mechanisms. Cell Host Microbe 15: 413–423 24721570 10.1016/j.chom.2014.03.006 PMC4020153

[embr202255664-bib-0059] De Luca A , Zelante T , D'Angelo C , Zagarella S , Fallarino F , Spreca A , Iannitti RG , Bonifazi P , Renauld J‐C , Bistoni F *et al* (2010) IL‐22 defines a novel immune pathway of antifungal resistance. Mucosal Immunol 3: 361–373 20445503 10.1038/mi.2010.22

[embr202255664-bib-0060] De Vadder F , Kovatcheva‐Datchary P , Goncalves D , Vinera J , Zitoun C , Duchampt A , Bäckhed F , Mithieux G (2014) Microbiota‐generated metabolites promote metabolic benefits via gut‐brain neural circuits. Cell 156: 84–96 24412651 10.1016/j.cell.2013.12.016

[embr202255664-bib-0061] De Vadder F , Kovatcheva‐Datchary P , Zitoun C , Duchampt A , Bäckhed F , Mithieux G (2016) Microbiota‐produced succinate improves glucose homeostasis via intestinal gluconeogenesis. Cell Metab 24: 151–157 27411015 10.1016/j.cmet.2016.06.013

[embr202255664-bib-0062] De Vadder F , Plessier F , Gautier‐Stein A , Mithieux G (2015) Vasoactive intestinal peptide is a local mediator in a gut‐brain neural axis activating intestinal gluconeogenesis. Neurogastroenterol Motil 27: 443–448 25586379 10.1111/nmo.12508

[embr202255664-bib-0063] Devlin AS , Marcobal A , Dodd D , Nayfach S , Plummer N , Meyer T , Pollard KS , Sonnenburg JL , Fischbach MA (2016) Modulation of a circulating uremic solute via rational genetic manipulation of the gut microbiota. Cell Host Microbe 20: 709–715 27916477 10.1016/j.chom.2016.10.021 PMC5159218

[embr202255664-bib-0064] Dey N , Wagner VE , Blanton LV , Cheng J , Fontana L , Haque R , Ahmed T , Gordon JI (2015) Regulators of gut motility revealed by a gnotobiotic model of diet‐microbiome interactions related to travel. Cell 163: 95–107 26406373 10.1016/j.cell.2015.08.059 PMC4583712

[embr202255664-bib-0065] Duan W , Ladenheim B , Cutler RG , Kruman II , Cadet JL , Mattson MP (2002) Dietary folate deficiency and elevated homocysteine levels endanger dopaminergic neurons in models of Parkinson's disease. J Neurochem 80: 101–110 11796748 10.1046/j.0022-3042.2001.00676.x

[embr202255664-bib-0066] Ducastel S , Touche V , Trabelsi M‐S , Boulinguiez A , Butruille L , Nawrot M , Peschard S , Chávez‐Talavera O , Dorchies E , Vallez E *et al* (2020) The nuclear receptor FXR inhibits glucagon‐like peptide‐1 secretion in response to microbiota‐derived short‐chain fatty acids. Sci Rep 10: 174 31932631 10.1038/s41598-019-56743-x PMC6957696

[embr202255664-bib-0067] Dumas M‐E , Rothwell AR , Hoyles L , Aranias T , Chilloux J , Calderari S , Noll EM , Péan N , Boulangé CL , Blancher C *et al* (2017) Microbial‐host co‐metabolites are prodromal markers predicting phenotypic heterogeneity in behavior, obesity, and impaired glucose tolerance. Cell Rep 20: 136–148 28683308 10.1016/j.celrep.2017.06.039 PMC5507771

[embr202255664-bib-0068] Eussen SJPM , Ueland PM , Vollset SE , Nygård O , Midttun Ø , Sulo G , Ulvik A , Meyer K , Pedersen ER , Tell GS (2015) Kynurenines as predictors of acute coronary events in the Hordaland Health Study. Int J Cardiol 189: 18–24 25885868 10.1016/j.ijcard.2015.03.413

[embr202255664-bib-0069] Fachi JL , Sécca C , Rodrigues PB , Mato FCP , Di Luccia B , Felipe JS , Pral LP , Rungue M , Rocha VM , Sato FT *et al* (2020) Acetate coordinates neutrophil and ILC3 responses against *C. difficile* through FFAR2. J Exp Med 217: jem.20190489 31876919 10.1084/jem.20190489 PMC7062529

[embr202255664-bib-0070] Fluhr L , Mor U , Kolodziejczyk AA , Dori‐Bachash M , Leshem A , Itav S , Cohen Y , Suez J , Zmora N , Moresi C *et al* (2021) Gut microbiota modulates weight gain in mice after discontinued smoke exposure. Nature 600: 713–719 34880502 10.1038/s41586-021-04194-8

[embr202255664-bib-0071] Fukata M , Chen A , Klepper A , Krishnareddy S , Vamadevan AS , Thomas LS , Xu R , Inoue H , Arditi M , Dannenberg AJ *et al* (2006) Cox‐2 is regulated by Toll‐like receptor‐4 (TLR4) signaling: role in proliferation and apoptosis in the intestine. Gastroenterology 131: 862–877 16952555 10.1053/j.gastro.2006.06.017 PMC2169292

[embr202255664-bib-0072] Fukata M , Chen A , Vamadevan AS , Cohen J , Breglio K , Krishnareddy S , Hsu D , Xu R , Harpaz N , Dannenberg AJ *et al* (2007) Toll‐like receptor‐4 promotes the development of colitis‐associated colorectal tumors. Gastroenterology 133: 1869–1881 18054559 10.1053/j.gastro.2007.09.008 PMC2180834

[embr202255664-bib-0073] Gantner BN , Simmons RM , Canavera SJ , Akira S , Underhill DM (2003) Collaborative induction of inflammatory responses by dectin‐1 and Toll‐like receptor 2. J Exp Med 197: 1107–1117 12719479 10.1084/jem.20021787 PMC2193968

[embr202255664-bib-0074] Gao F , Lv Y‐W , Long J , Chen J‐M , He J‐M , Ruan X‐Z , Zhu H‐B (2019) Butyrate improves the metabolic disorder and gut microbiome dysbiosis in mice induced by a high‐fat diet. Front Pharmacol 10: 1040 31607907 10.3389/fphar.2019.01040 PMC6761375

[embr202255664-bib-0075] Gao Z , Yin J , Zhang J , Ward RE , Martin RJ , Lefevre M , Cefalu WT , Ye J (2009) Butyrate improves insulin sensitivity and increases energy expenditure in mice. Diabetes 58: 1509–1517 19366864 10.2337/db08-1637 PMC2699871

[embr202255664-bib-0076] Goodrich JK , Waters JL , Poole AC , Sutter JL , Koren O , Blekhman R , Beaumont M , Van Treuren W , Knight R , Bell JT *et al* (2014) Human genetics shape the gut microbiome. Cell 159: 789–799 25417156 10.1016/j.cell.2014.09.053 PMC4255478

[embr202255664-bib-0077] Grosheva I , Zheng D , Levy M , Polansky O , Lichtenstein A , Golani O , Dori‐Bachash M , Moresi C , Shapiro H , Del Mare‐Roumani S *et al* (2020) High‐throughput screen identifies host and microbiota regulators of intestinal barrier function. Gastroenterology 159: 1807–1823 32653496 10.1053/j.gastro.2020.07.003

[embr202255664-bib-0078] Guzior DV , Quinn RA (2021) Review: microbial transformations of human bile acids. Microbiome 9: 140 34127070 10.1186/s40168-021-01101-1 PMC8204491

[embr202255664-bib-0079] Hall JA , Bouladoux N , Sun CM , Wohlfert EA , Blank RB , Zhu Q , Grigg ME , Berzofsky JA , Belkaid Y (2008) Commensal DNA limits regulatory T cell conversion and is a natural adjuvant of intestinal immune responses. Immunity 29: 637–649 18835196 10.1016/j.immuni.2008.08.009 PMC2712925

[embr202255664-bib-0080] Hang S , Paik D , Yao L , Kim E , Trinath J , Lu J , Ha S , Nelson BN , Kelly SP , Wu L *et al* (2019) Bile acid metabolites control TH17 and Treg cell differentiation. Nature 576: 143–148 31776512 10.1038/s41586-019-1785-z PMC6949019

[embr202255664-bib-0081] Hao F , Tian M , Zhang X , Jin X , Jiang Y , Sun X , Wang Y , Peng P , Liu J , Xia C *et al* (2021) Butyrate enhances CPT1A activity to promote fatty acid oxidation and iTreg differentiation. Proc Natl Acad Sci U S A 118: e2014681118 34035164 10.1073/pnas.2014681118 PMC8179238

[embr202255664-bib-0082] Hayashi A , Nagao‐Kitamoto H , Kitamoto S , Kim CH , Kamada N (2021a) The butyrate‐producing bacterium clostridium butyricum suppresses *Clostridioides difficile* infection via neutrophil‐ and antimicrobial cytokine‐dependent but GPR43/109a‐independent mechanisms. J Immunol 206: 1576–1585 33597149 10.4049/jimmunol.2000353 PMC7980534

[embr202255664-bib-0083] Hayashi T , Yamashita T , Takahashi T , Tabata T , Watanabe H , Gotoh Y , Shinohara M , Kami K , Tanaka H , Matsumoto K *et al* (2021b) Uncovering the role of gut microbiota in amino acid metabolic disturbances in heart failure through metagenomic analysis. Front Cardiovasc Med 8: 789325 34912870 10.3389/fcvm.2021.789325 PMC8667331

[embr202255664-bib-0084] van der Hee B , Wells JM (2021) Microbial regulation of host physiology by short‐chain fatty acids. Trends Microbiol 29: 700–712 33674141 10.1016/j.tim.2021.02.001

[embr202255664-bib-0085] Hehemann J‐H , Correc G , Barbeyron T , Helbert W , Czjzek M , Michel G (2010) Transfer of carbohydrate‐active enzymes from marine bacteria to Japanese gut microbiota. Nature 464: 908–912 20376150 10.1038/nature08937

[embr202255664-bib-0086] Henao‐Mejia J , Elinav E , Jin C , Hao L , Mehal WZ , Strowig T , Thaiss CA , Kau AL , Eisenbarth SC , Jurczak MJ *et al* (2012) Inflammasome‐mediated dysbiosis regulates progression of NAFLD and obesity. Nature 482: 179–185 22297845 10.1038/nature10809 PMC3276682

[embr202255664-bib-0087] Hendrikx T , Duan Y , Wang Y , Oh J‐H , Alexander LM , Huang W , Stärkel P , Ho SB , Gao B , Fiehn O *et al* (2019) Bacteria engineered to produce IL‐22 in intestine induce expression of REG3G to reduce ethanol‐induced liver disease in mice. Gut 68: 1504–1515 30448775 10.1136/gutjnl-2018-317232 PMC6387784

[embr202255664-bib-0088] Hoebe K , Georgel P , Rutschmann S , Du X , Mudd S , Crozat K , Sovath S , Shamel L , Hartung T , Zähringer U *et al* (2005) CD36 is a sensor of diacylglycerides. Nature 433: 523–527 15690042 10.1038/nature03253

[embr202255664-bib-0089] Hsu D , Fukata M , Hernandez YG , Sotolongo JP , Goo T , Maki J , Hayes LA , Ungaro RC , Chen A , Breglio KJ *et al* (2010) Toll‐like receptor 4 differentially regulates epidermal growth factor‐related growth factors in response to intestinal mucosal injury. Lab Invest 90: 1295–1305 20498653 10.1038/labinvest.2010.100 PMC10631458

[embr202255664-bib-0090] Induri SNR , Kansara P , Thomas SC , Xu F , Saxena D , Li X (2022) The gut microbiome, metformin, and aging. Annu Rev Pharmacol Toxicol 62: 85–108 34449247 10.1146/annurev-pharmtox-051920-093829

[embr202255664-bib-0091] Iqbal J , Hussain MM (2009) Intestinal lipid absorption. Am J Physiol Endocrinol Metab 296: E1183–E1194 19158321 10.1152/ajpendo.90899.2008 PMC2692399

[embr202255664-bib-0092] Isobe J , Maeda S , Obata Y , Iizuka K , Nakamura Y , Fujimura Y , Kimizuka T , Hattori K , Kim Y‐G , Morita T *et al* (2020) Commensal‐bacteria‐derived butyrate promotes the T‐cell‐independent IgA response in the colon. Int Immunol 32: 243–258 31858119 10.1093/intimm/dxz078

[embr202255664-bib-0093] Ivanov II , Atarashi K , Manel N , Brodie EL , Shima T , Karaoz U , Wei D , Goldfarb KC , Santee CA , Lynch SV *et al* (2009) Induction of intestinal Th17 cells by segmented filamentous bacteria. Cell 139: 485–498 19836068 10.1016/j.cell.2009.09.033 PMC2796826

[embr202255664-bib-0094] Jamka M , Kokot M , Kaczmarek N , Bermagambetova S , Nowak JK , Walkowiak J (2021) The effect of sodium butyrate enemas compared with placebo on disease activity, endoscopic scores, and histological and inflammatory parameters in inflammatory bowel diseases: a systematic review of randomised controlled trials. Complement Med Res 28: 344–356 33352566 10.1159/000512952

[embr202255664-bib-0095] Jiang C , Xie C , Lv Y , Li J , Krausz KW , Shi J , Brocker CN , Desai D , Amin SG , Bisson WH *et al* (2015) Intestine‐selective farnesoid X receptor inhibition improves obesity‐related metabolic dysfunction. Nat Commun 6: 10166 26670557 10.1038/ncomms10166 PMC4682112

[embr202255664-bib-0096] Jiang Z , Georgel P , Du X , Shamel L , Sovath S , Mudd S , Huber M , Kalis C , Keck S , Galanos C *et al* (2005) CD14 is required for MyD88‐independent LPS signaling. Nat Immunol 6: 565–570 15895089 10.1038/ni1207

[embr202255664-bib-0097] Jie Z , Xia H , Zhong S‐L , Feng Q , Li S , Liang S , Zhong H , Liu Z , Gao Y , Zhao H *et al* (2017) The gut microbiome in atherosclerotic cardiovascular disease. Nat Commun 8: 845 29018189 10.1038/s41467-017-00900-1 PMC5635030

[embr202255664-bib-0098] Johnson EL , Heaver SL , Waters JL , Kim BI , Bretin A , Goodman AL , Gewirtz AT , Worgall TS , Ley RE (2020) Sphingolipids produced by gut bacteria enter host metabolic pathways impacting ceramide levels. Nat Commun 11: 2471 32424203 10.1038/s41467-020-16274-w PMC7235224

[embr202255664-bib-0099] Keane C , Tilley D , Cunningham A , Smolenski A , Kadioglu A , Cox D , Jenkinson HF , Kerrigan SW (2010) Invasive *Streptococcus pneumoniae* trigger platelet activation via Toll‐like receptor 2. J Thromb Haemost 8: 2757–2765 20946179 10.1111/j.1538-7836.2010.04093.x

[embr202255664-bib-0100] Kim MH , Kang SG , Park JH , Yanagisawa M , Kim CH (2013) Short‐chain fatty acids activate GPR41 and GPR43 on intestinal epithelial cells to promote inflammatory responses in mice. Gastroenterology 145: 396–406 23665276 10.1053/j.gastro.2013.04.056

[embr202255664-bib-0101] Kinnebrew MA , Buffie CG , Diehl GE , Zenewicz LA , Leiner I , Hohl TM , Flavell RA , Littman DR , Pamer EG (2012) Interleukin 23 production by intestinal CD103(+)CD11b(+) dendritic cells in response to bacterial flagellin enhances mucosal innate immune defense. Immunity 36: 276–287 22306017 10.1016/j.immuni.2011.12.011 PMC3288454

[embr202255664-bib-0102] Koeth RA , Wang Z , Levison BS , Buffa JA , Org E , Sheehy BT , Britt EB , Fu X , Wu Y , Li L *et al* (2013) Intestinal microbiota metabolism of L‐carnitine, a nutrient in red meat, promotes atherosclerosis. Nat Med 19: 576–585 23563705 10.1038/nm.3145 PMC3650111

[embr202255664-bib-0103] Koh A , Bäckhed F (2020) From association to causality: the role of the gut microbiota and its functional products on host metabolism. Mol Cell 78: 584–596 32234490 10.1016/j.molcel.2020.03.005

[embr202255664-bib-0104] Kolodziejczyk AA , Federici S , Zmora N , Mohapatra G , Dori‐Bachash M , Hornstein S , Leshem A , Reuveni D , Zigmond E , Tobar A *et al* (2020) Acute liver failure is regulated by MYC‐ and microbiome‐dependent programs. Nat Med 26: 1899–1911 33106666 10.1038/s41591-020-1102-2

[embr202255664-bib-0105] Kolodziejczyk AA , Zheng D , Elinav E (2019) Diet–microbiota interactions and personalized nutrition. Nat Rev Microbiol 17: 742–753 31541197 10.1038/s41579-019-0256-8

[embr202255664-bib-0106] Korpe PS , Petri WA Jr (2012) Environmental enteropathy: critical implications of a poorly understood condition. Trends Mol Med 18: 328–336 22633998 10.1016/j.molmed.2012.04.007 PMC3372657

[embr202255664-bib-0107] Krautkramer KA , Fan J , Bäckhed F (2021) Gut microbial metabolites as multi‐kingdom intermediates. Nat Rev Microbiol 19: 77–94 32968241 10.1038/s41579-020-0438-4

[embr202255664-bib-0108] Lamas B , Richard ML , Leducq V , Pham H‐P , Michel M‐L , Da Costa G , Bridonneau C , Jegou S , Hoffmann TW , Natividad JM *et al* (2016) CARD9 impacts colitis by altering gut microbiota metabolism of tryptophan into aryl hydrocarbon receptor ligands. Nat Med 22: 598–605 27158904 10.1038/nm.4102 PMC5087285

[embr202255664-bib-0109] Laurans L , Venteclef N , Haddad Y , Chajadine M , Alzaid F , Metghalchi S , Sovran B , Denis RGP , Dairou J , Cardellini M *et al* (2018) Genetic deficiency of indoleamine 2,3‐dioxygenase promotes gut microbiota‐mediated metabolic health. Nat Med 24: 1113–1120 29942089 10.1038/s41591-018-0060-4

[embr202255664-bib-0110] Lee Y‐S , Kim T‐Y , Kim Y , Lee S‐H , Kim S , Kang SW , Yang J‐Y , Baek I‐J , Sung YH , Park Y‐Y *et al* (2018) Microbiota‐derived lactate accelerates intestinal stem‐cell‐mediated epithelial development. Cell Host Microbe 24: 833–846 30543778 10.1016/j.chom.2018.11.002

[embr202255664-bib-0111] León‐Mimila P , Villamil‐Ramírez H , Li XS , Shih DM , Hui ST , Ocampo‐Medina E , López‐Contreras B , Morán‐Ramos S , Olivares‐Arevalo M , Grandini‐Rosales P *et al* (2021) Trimethylamine N‐oxide levels are associated with NASH in obese subjects with type 2 diabetes. Diabetes Metab 47: 101183 32791310 10.1016/j.diabet.2020.07.010 PMC8018562

[embr202255664-bib-0112] Lev‐Sagie A , Goldman‐Wohl D , Cohen Y , Dori‐Bachash M , Leshem A , Mor U , Strahilevitz J , Moses AE , Shapiro H , Yagel S *et al* (2019) Vaginal microbiome transplantation in women with intractable bacterial vaginosis. Nat Med 25: 1500–1504 31591599 10.1038/s41591-019-0600-6

[embr202255664-bib-0113] Levy M , Thaiss CA , Zeevi D , Dohnalová L , Zilberman‐Schapira G , Mahdi JA , David E , Savidor A , Korem T , Herzig Y *et al* (2015) Microbiota‐modulated metabolites shape the intestinal microenvironment by regulating NLRP6 inflammasome signaling. Cell 163: 1428–1443 26638072 10.1016/j.cell.2015.10.048 PMC5665753

[embr202255664-bib-0114] Li B , Li L , Li M , Lam SM , Wang G , Wu Y , Zhang H , Niu C , Zhang X , Liu X *et al* (2019) Microbiota depletion impairs thermogenesis of brown adipose tissue and browning of white adipose tissue. Cell Rep 26: 2720–2737 30840893 10.1016/j.celrep.2019.02.015

[embr202255664-bib-0115] Li F , Jiang C , Krausz KW , Li Y , Albert I , Hao H , Fabre KM , Mitchell JB , Patterson AD , Gonzalez FJ (2013) Microbiome remodelling leads to inhibition of intestinal farnesoid X receptor signalling and decreased obesity. Nat Commun 4: 2384 24064762 10.1038/ncomms3384 PMC6595219

[embr202255664-bib-0116] Li G , Xie C , Lu S , Nichols RG , Tian Y , Li L , Patel D , Ma Y , Brocker CN , Yan T *et al* (2017) Intermittent fasting promotes white adipose browning and decreases obesity by shaping the gut microbiota. Cell Metab 26: 672–685 28918936 10.1016/j.cmet.2017.08.019 PMC5668683

[embr202255664-bib-0117] Li Z , Yi C‐X , Katiraei S , Kooijman S , Zhou E , Chung CK , Gao Y , van den Heuvel JK , Meijer OC , Berbée JFP *et al* (2018) Butyrate reduces appetite and activates brown adipose tissue via the gut‐brain neural circuit. Gut 67: 1269–1279 29101261 10.1136/gutjnl-2017-314050

[embr202255664-bib-0118] Liang Z , Dong Z , Guo M , Shen Z , Yin D , Hu S , Hai X (2019) Trimethylamine N‐oxide as a risk marker for ischemic stroke in patients with atrial fibrillation. J Biochem Mol Toxicol 33: e22246 30370581 10.1002/jbt.22246

[embr202255664-bib-0119] Lobo AR , Gaievski EHS , De Carli E , Alvares EP , Colli C (2014) Fructo‐oligosaccharides and iron bioavailability in anaemic rats: the effects on iron species distribution, ferroportin‐1 expression, crypt bifurcation and crypt cell proliferation in the caecum. Br J Nutr 112: 1286–1295 25192308 10.1017/S0007114514002165

[embr202255664-bib-0120] Louis P , Flint HJ (2017) Formation of propionate and butyrate by the human colonic microbiota. Environ Microbiol 19: 29–41 27928878 10.1111/1462-2920.13589

[embr202255664-bib-0121] Lundberg R , Toft MF , Metzdorff SB , Hansen CHF , Licht TR , Bahl MI , Hansen AK (2020) Human microbiota‐transplanted C57BL/6 mice and offspring display reduced establishment of key bacteria and reduced immune stimulation compared to mouse microbiota‐transplantation. Sci Rep 10: 7805 32385373 10.1038/s41598-020-64703-z PMC7211022

[embr202255664-bib-0122] Luu M , Weigand K , Wedi F , Breidenbend C , Leister H , Pautz S , Adhikary T , Visekruna A (2018) Regulation of the effector function of CD8+ T cells by gut microbiota‐derived metabolite butyrate. Sci Rep 8: 14430 30258117 10.1038/s41598-018-32860-x PMC6158259

[embr202255664-bib-0123] Macho Fernandez E , Valenti V , Rockel C , Hermann C , Pot B , Boneca IG , Grangette C (2011) Anti‐inflammatory capacity of selected lactobacilli in experimental colitis is driven by NOD2‐mediated recognition of a specific peptidoglycan‐derived muropeptide. Gut 60: 1050–1059 21471573 10.1136/gut.2010.232918

[embr202255664-bib-0124] Macia L , Tan J , Vieira AT , Leach K , Stanley D , Luong S , Maruya M , Ian McKenzie C , Hijikata A , Wong C *et al* (2015) Metabolite‐sensing receptors GPR43 and GPR109A facilitate dietary fibre‐induced gut homeostasis through regulation of the inflammasome. Nat Commun 6: 6734 25828455 10.1038/ncomms7734

[embr202255664-bib-0125] Mandaliya DK , Patel S , Seshadri S (2021) The combinatorial effect of acetate and propionate on high‐fat diet induced diabetic inflammation or metaflammation and T cell polarization. Inflammation 44: 68–79 32978698 10.1007/s10753-020-01309-7

[embr202255664-bib-0126] Marques FZ , Nelson E , Chu P‐Y , Horlock D , Fiedler A , Ziemann M , Tan JK , Kuruppu S , Rajapakse NW , El‐Osta A *et al* (2017) High‐fiber diet and acetate supplementation change the gut microbiota and prevent the development of hypertension and heart failure in hypertensive mice. Circulation 135: 964–977 27927713 10.1161/CIRCULATIONAHA.116.024545

[embr202255664-bib-0127] Martinez‐Guryn K , Hubert N , Frazier K , Urlass S , Musch MW , Ojeda P , Pierre JF , Miyoshi J , Sontag TJ , Cham CM *et al* (2018) Small intestine microbiota regulate host digestive and absorptive adaptive responses to dietary lipids. Cell Host Microbe 23: 458–469 29649441 10.1016/j.chom.2018.03.011 PMC5912695

[embr202255664-bib-0128] Mazagova M , Wang L , Anfora AT , Wissmueller M , Lesley SA , Miyamoto Y , Eckmann L , Dhungana S , Pathmasiri W , Sumner S *et al* (2015) Commensal microbiota is hepatoprotective and prevents liver fibrosis in mice. FASEB J 29: 1043–1055 25466902 10.1096/fj.14-259515 PMC4422368

[embr202255664-bib-0129] Mazmanian SK , Liu CH , Tzianabos AO , Kasper DL (2005) An immunomodulatory molecule of symbiotic bacteria directs maturation of the host immune system. Cell 122: 107–118 16009137 10.1016/j.cell.2005.05.007

[embr202255664-bib-0130] Mazmanian SK , Round JL , Kasper DL (2008) A microbial symbiosis factor prevents intestinal inflammatory disease. Nature 453: 620–625 18509436 10.1038/nature07008

[embr202255664-bib-0131] McFall‐Ngai M (2007) Adaptive immunity: care for the community. Nature 445: 153 17215830 10.1038/445153a

[embr202255664-bib-0132] McFarland LV (2014) Use of probiotics to correct dysbiosis of normal microbiota following disease or disruptive events: a systematic review. BMJ Open 4: e005047 10.1136/bmjopen-2014-005047 PMC4156804 25157183

[embr202255664-bib-0133] Miani M , Le Naour J , Waeckel‐Enée E , Verma SC , Straube M , Emond P , Ryffel B , van Endert P , Sokol H , Diana J (2018) Gut microbiota‐stimulated innate lymphoid cells support β‐defensin 14 expression in pancreatic endocrine cells, preventing autoimmune diabetes. Cell Metab 28: 557–572 30017352 10.1016/j.cmet.2018.06.012

[embr202255664-bib-0134] Mocanu V , Zhang Z , Deehan EC , Kao DH , Hotte N , Karmali S , Birch DW , Samarasinghe KK , Walter J , Madsen KL (2021) Fecal microbial transplantation and fiber supplementation in patients with severe obesity and metabolic syndrome: a randomized double‐blind, placebo‐controlled phase 2 trial. Nat Med 27: 1272–1279 34226737 10.1038/s41591-021-01399-2

[embr202255664-bib-0135] Muller PA , Schneeberger M , Matheis F , Wang P , Kerner Z , Ilanges A , Pellegrino K , Del Mármol J , Castro TBR , Furuichi M *et al* (2020) Microbiota modulate sympathetic neurons via a gut‐brain circuit. Nature 583: 441–446 32641826 10.1038/s41586-020-2474-7 PMC7367767

[embr202255664-bib-0136] Needham BD , Kaddurah‐Daouk R , Mazmanian SK (2020) Gut microbial molecules in behavioural and neurodegenerative conditions. Nat Rev Neurosci 21: 717–731 33067567 10.1038/s41583-020-00381-0

[embr202255664-bib-0137] Neu AT , Allen EE , Roy K (2021) Defining and quantifying the core microbiome: challenges and prospects. Proc Natl Acad Sci U S A 118: e2104429118 34862327 10.1073/pnas.2104429118 PMC8713806

[embr202255664-bib-0138] Nie J , Xie L , Zhao B‐X , Li Y , Qiu B , Zhu F , Li G‐F , He M , Wang Y , Wang B *et al* (2018) Serum trimethylamine N‐oxide concentration is positively associated with first stroke in hypertensive patients. Stroke 49: 2021–2028 30354996 10.1161/STROKEAHA.118.021997

[embr202255664-bib-0139] Normand S , Delanoye‐Crespin A , Bressenot A , Huot L , Grandjean T , Peyrin‐Biroulet L , Lemoine Y , Hot D , Chamaillard M (2011) Nod‐like receptor pyrin domain‐containing protein 6 (NLRP6) controls epithelial self‐renewal and colorectal carcinogenesis upon injury. Proc Natl Acad Sci U S A 108: 9601–9606 21593405 10.1073/pnas.1100981108 PMC3111299

[embr202255664-bib-0140] Ogura Y , Lala S , Xin W , Smith E , Dowds TA , Chen FF , Zimmermann E , Tretiakova M , Cho JH , Hart J *et al* (2003) Expression of NOD2 in Paneth cells: a possible link to Crohn's ileitis. Gut 52: 1591–1597 14570728 10.1136/gut.52.11.1591 PMC1773866

[embr202255664-bib-0141] Oh SF , Praveena T , Song H , Yoo J‐S , Jung D‐J , Erturk‐Hasdemir D , Hwang YS , Lee CC , Le Nours J , Kim H *et al* (2021) Host immunomodulatory lipids created by symbionts from dietary amino acids. Nature 600: 302–307 34759313 10.1038/s41586-021-04083-0 PMC8999822

[embr202255664-bib-0142] Ohira H , Fujioka Y , Katagiri C , Mamoto R , Aoyama‐Ishikawa M , Amako K , Izumi Y , Nishiumi S , Yoshida M , Usami M *et al* (2013) Butyrate attenuates inflammation and lipolysis generated by the interaction of adipocytes and macrophages. J Atheroscler Thromb 20: 425–442 23470566 10.5551/jat.15065

[embr202255664-bib-0143] Okada T , Fukuda S , Hase K , Nishiumi S , Izumi Y , Yoshida M , Hagiwara T , Kawashima R , Yamazaki M , Oshio T *et al* (2013) Microbiota‐derived lactate accelerates colon epithelial cell turnover in starvation‐refed mice. Nat Commun 4: 1654 23552069 10.1038/ncomms2668

[embr202255664-bib-0144] Okla M , Zaher W , Alfayez M , Chung S (2018) Inhibitory effects of toll‐like receptor 4, NLRP3 inflammasome, and interleukin‐1β on white adipocyte browning. Inflammation 41: 626–642 29264745 10.1007/s10753-017-0718-y PMC6066287

[embr202255664-bib-0145] Paeslack N , Mimmler M , Becker S , Gao Z , Khuu MP , Mann A , Malinarich F , Regen T , Reinhardt C (2022) Microbiota‐derived tryptophan metabolites in vascular inflammation and cardiovascular disease. Amino Acids 10.1007/s00726-022-03161-5 PMC9641817 35451695

[embr202255664-bib-0146] Park J , Goergen CJ , HogenEsch H , Kim CH (2016) Chronically elevated levels of short‐chain fatty acids induce T cell‐mediated ureteritis and hydronephrosis. J Immunol 196: 2388–2400 26819206 10.4049/jimmunol.1502046 PMC4761537

[embr202255664-bib-0147] Parséus A , Sommer N , Sommer F , Caesar R , Molinaro A , Ståhlman M , Greiner TU , Perkins R , Bäckhed F (2017) Microbiota‐induced obesity requires farnesoid X receptor. Gut 66: 429–437 26740296 10.1136/gutjnl-2015-310283 PMC5534765

[embr202255664-bib-0148] Pathak P , Xie C , Nichols RG , Ferrell JM , Boehme S , Krausz KW , Patterson AD , Gonzalez FJ , Chiang JYL (2018) Intestine farnesoid X receptor agonist and the gut microbiota activate G‐protein bile acid receptor‐1 signaling to improve metabolism. Hepatology 68: 1574–1588 29486523 10.1002/hep.29857 PMC6111007

[embr202255664-bib-0149] Podolsky DK , Gerken G , Eyking A , Cario E (2009) Colitis‐associated variant of TLR2 causes impaired mucosal repair because of TFF3 deficiency. Gastroenterology 137: 209–220 19303021 10.1053/j.gastro.2009.03.007 PMC2812790

[embr202255664-bib-0150] Pott J , Hornef M (2012) Innate immune signalling at the intestinal epithelium in homeostasis and disease. EMBO Rep 13: 684–698 22801555 10.1038/embor.2012.96 PMC3410395

[embr202255664-bib-0151] Qi X , Yun C , Sun L , Xia J , Wu Q , Wang Y , Wang L , Zhang Y , Liang X , Wang L *et al* (2019) Gut microbiota‐bile acid‐interleukin‐22 axis orchestrates polycystic ovary syndrome. Nat Med 25: 1225–1233 31332392 10.1038/s41591-019-0509-0 PMC7376369

[embr202255664-bib-0152] Qin J , Li Y , Cai Z , Li S , Zhu J , Zhang F , Liang S , Zhang W , Guan Y , Shen D *et al* (2012) A metagenome‐wide association study of gut microbiota in type 2 diabetes. Nature 490: 55–60 23023125 10.1038/nature11450

[embr202255664-bib-0153] Quigley EMM , Murray JA , Pimentel M (2020) AGA clinical practice update on small intestinal bacterial overgrowth: expert review. Gastroenterology 159: 1526–1532 32679220 10.1053/j.gastro.2020.06.090

[embr202255664-bib-0154] Quinn RA , Melnik AV , Vrbanac A , Fu T , Patras KA , Christy MP , Bodai Z , Belda‐Ferre P , Tripathi A , Chung LK *et al* (2020) Global chemical effects of the microbiome include new bile‐acid conjugations. Nature 579: 123–129 32103176 10.1038/s41586-020-2047-9 PMC7252668

[embr202255664-bib-0155] Rakoff‐Nahoum S , Paglino J , Eslami‐Varzaneh F , Edberg S , Medzhitov R (2004) Recognition of commensal microflora by toll‐like receptors is required for intestinal homeostasis. Cell 118: 229–241 15260992 10.1016/j.cell.2004.07.002

[embr202255664-bib-0156] Rein M , Ben‐Yacov O , Godneva A , Shilo S , Zmora N , Kolobkov D , Cohen‐Dolev N , Wolf B‐C , Kosower N , Lotan‐Pompan M *et al* (2022) Effects of personalized diets by prediction of glycemic responses on glycemic control and metabolic health in newly diagnosed T2DM: a randomized dietary intervention pilot trial. BMC Med 20: 56 35135549 10.1186/s12916-022-02254-y PMC8826661

[embr202255664-bib-0157] Ridaura VK , Faith JJ , Rey FE , Cheng J , Duncan AE , Kau AL , Griffin NW , Lombard V , Henrissat B , Bain JR *et al* (2013) Gut microbiota from twins discordant for obesity modulate metabolism in mice. Science 341: 1241214 24009397 10.1126/science.1241214 PMC3829625

[embr202255664-bib-0158] Romano KA , Martinez‐Del Campo A , Kasahara K , Chittim CL , Vivas EI , Amador‐Noguez D , Balskus EP , Rey FE (2017) Metabolic, epigenetic, and transgenerational effects of gut bacterial choline consumption. Cell Host Microbe 22: 279–290 28844887 10.1016/j.chom.2017.07.021 PMC5599363

[embr202255664-bib-0159] Rosario D , Bidkhori G , Lee S , Bedarf J , Hildebrand F , Le Chatelier E , Uhlen M , Ehrlich SD , Proctor G , Wüllner U *et al* (2021) Systematic analysis of gut microbiome reveals the role of bacterial folate and homocysteine metabolism in Parkinson's disease. Cell Rep 34: 108807 33657381 10.1016/j.celrep.2021.108807

[embr202255664-bib-0160] Roshanravan N , Mahdavi R , Alizadeh E , Jafarabadi M , Hedayati M , Ghavami A , Alipour S , Alamdari N , Barati M , Ostadrahimi A (2017) Effect of butyrate and inulin supplementation on glycemic status, lipid profile and glucagon‐like peptide 1 level in patients with type 2 diabetes: a randomized double‐blind, placebo‐controlled trial. Horm Metab Res 49: 886–891 28962046 10.1055/s-0043-119089

[embr202255664-bib-0161] Rossi T , Vergara D , Fanini F , Maffia M , Bravaccini S , Pirini F (2020) Microbiota‐derived metabolites in tumor progression and metastasis. Int J Mol Sci 21: 5786 32806665 10.3390/ijms21165786 PMC7460823

[embr202255664-bib-0162] Roth GA , Mensah GA , Johnson CO , Giovanni A , Enrico A , Baddour LM , Barengo NC , Beaton AZ , Benjamin EJ , Benziger CP *et al* (2020) Global burden of cardiovascular diseases and risk factors, 1990–2019. J Am Coll Cardiol 76: 2982–3021 33309175 10.1016/j.jacc.2020.11.010 PMC7755038

[embr202255664-bib-0163] Round JL , Lee SM , Li J , Tran G , Jabri B , Chatila TA , Mazmanian SK (2011) The Toll‐like receptor 2 pathway establishes colonization by a commensal of the human microbiota. Science 332: 974–977 21512004 10.1126/science.1206095 PMC3164325

[embr202255664-bib-0164] Roy S , Yuzefpolskaya M , Nandakumar R , Colombo PC , Demmer RT (2020) Plasma Trimethylamine‐N‐oxide and impaired glucose regulation: Results from The Oral Infections, Glucose Intolerance and Insulin Resistance Study (ORIGINS). PLoS One 15: e0227482 31940332 10.1371/journal.pone.0227482 PMC6961885

[embr202255664-bib-0165] Salminen S , Collado MC , Endo A , Hill C , Lebeer S , Quigley EMM , Sanders ME , Shamir R , Swann JR , Szajewska H *et al* (2021) The International Scientific Association of Probiotics and Prebiotics (ISAPP) consensus statement on the definition and scope of postbiotics. Nat Rev Gastroenterol Hepatol 18: 649–667 33948025 10.1038/s41575-021-00440-6 PMC8387231

[embr202255664-bib-0166] Sampson TR , Challis C , Jain N , Moiseyenko A , Ladinsky MS , Shastri GG , Thron T , Needham BD , Horvath I , Debelius JW *et al* (2020) A gut bacterial amyloid promotes α‐synuclein aggregation and motor impairment in mice. Elife 9: e53111 32043464 10.7554/eLife.53111 PMC7012599

[embr202255664-bib-0167] Samuel BS , Shaito A , Motoike T , Rey FE , Backhed F , Manchester JK , Hammer RE , Williams SC , Crowley J , Yanagisawa M *et al* (2008) Effects of the gut microbiota on host adiposity are modulated by the short‐chain fatty‐acid binding G protein‐coupled receptor, Gpr41. Proc Natl Acad Sci U S A 105: 16767–16772 18931303 10.1073/pnas.0808567105 PMC2569967

[embr202255664-bib-0168] dos Santos EF , Busanello ENB , Miglioranza A , Zanatta A , Barchak AG , Vargas CR , Saute J , Rosa C , Carrion MJ , Camargo D *et al* (2009) Evidence that folic acid deficiency is a major determinant of hyperhomocysteinemia in Parkinson's disease. Metab Brain Dis 24: 257–269 19294496 10.1007/s11011-009-9139-4

[embr202255664-bib-0169] Sato FT , Yap YA , Crisma AR , Portovedo M , Murata GM , Hirabara SM , Ribeiro WR , Marcantonio Ferreira C , Cruz MM , Pereira JNB *et al* (2020) Tributyrin attenuates metabolic and inflammatory changes associated with obesity through a GPR109A‐dependent mechanism. Cell 9: 2007 10.3390/cells9092007 PMC7563536 32882837

[embr202255664-bib-0170] Sato Y , Atarashi K , Plichta DR , Arai Y , Sasajima S , Kearney SM , Suda W , Takeshita K , Sasaki T , Okamoto S *et al* (2021) Novel bile acid biosynthetic pathways are enriched in the microbiome of centenarians. Nature 599: 458–464 34325466 10.1038/s41586-021-03832-5

[embr202255664-bib-0171] Scheppach W , German‐Austrian SCFA Study Group (1996) Treatment of distal ulcerative colitis with short‐chain fatty acid enemas a placebo‐controlled trial. Dig Dis Sci 41: 2254–2259 8943981 10.1007/BF02071409

[embr202255664-bib-0172] Scheppach W , Sommer H , Kirchner T , Paganelli GM , Bartram P , Christl S , Richter F , Dusel G , Kasper H (1992) Effect of butyrate enemas on the colonic mucosa in distal ulcerative colitis. Gastroenterology 103: 51–56 1612357 10.1016/0016-5085(92)91094-k

[embr202255664-bib-0173] Scholz‐Ahrens KE , Schrezenmeir J (2002) Inulin, oligofructose and mineral metabolism — experimental data and mechanism. Br J Nutr 87: S179 12088516 10.1079/BJNBJN/2002535

[embr202255664-bib-0174] Schupack DA , Mars RAT , Voelker DH , Abeykoon JP , Kashyap PC (2022) The promise of the gut microbiome as part of individualized treatment strategies. Nat Rev Gastroenterol Hepatol 19: 7–25 34453142 10.1038/s41575-021-00499-1 PMC8712374

[embr202255664-bib-0175] Seekatz AM , Theriot CM , Rao K , Chang Y‐M , Freeman AE , Kao JY , Young VB (2018) Restoration of short chain fatty acid and bile acid metabolism following fecal microbiota transplantation in patients with recurrent Clostridium difficile infection. Anaerobe 53: 64–73 29654837 10.1016/j.anaerobe.2018.04.001 PMC6185828

[embr202255664-bib-0176] Senthong V , Li XS , Hudec T , Coughlin J , Wu Y , Levison B , Wang Z , Hazen SL , Tang WHW (2016) Plasma trimethylamine N‐Oxide, a gut microbe‐generated phosphatidylcholine metabolite, is associated with atherosclerotic burden. J Am Coll Cardiol 67: 2620–2628 27256833 10.1016/j.jacc.2016.03.546 PMC4893167

[embr202255664-bib-0177] Serino M , Luche E , Gres S , Baylac A , Bergé M , Cenac C , Waget A , Klopp P , Iacovoni J , Klopp C *et al* (2012) Metabolic adaptation to a high‐fat diet is associated with a change in the gut microbiota. Gut 61: 543–553 22110050 10.1136/gutjnl-2011-301012 PMC3292714

[embr202255664-bib-0178] Sharon G , Cruz NJ , Kang D‐W , Gandal MJ , Wang B , Kim Y‐M , Zink EM , Casey CP , Taylor BC , Lane CJ *et al* (2019) Human gut microbiota from autism spectrum disorder promote behavioral symptoms in mice. Cell 177: 1600–1618 31150625 10.1016/j.cell.2019.05.004 PMC6993574

[embr202255664-bib-0179] Singh DP , Borse SP , Nivsarkar M (2017) Overcoming the exacerbating effects of ranitidine on NSAID‐induced small intestinal toxicity with quercetin: providing a complete GI solution. Chem Biol Interact 272: 53–64 28400101 10.1016/j.cbi.2017.04.006

[embr202255664-bib-0180] Singh N , Gurav A , Sivaprakasam S , Brady E , Padia R , Shi H , Thangaraju M , Prasad PD , Manicassamy S , Munn DH *et al* (2014) Activation of Gpr109a, receptor for niacin and the commensal metabolite butyrate, suppresses colonic inflammation and carcinogenesis. Immunity 40: 128–139 24412617 10.1016/j.immuni.2013.12.007 PMC4305274

[embr202255664-bib-0181] Smith PM , Howitt MR , Panikov N , Michaud M , Gallini CA , Bohlooly‐Y M , Glickman JN , Garrett WS (2013) The microbial metabolites, short‐chain fatty acids, regulate colonic Treg cell homeostasis. Science 341: 569–573 23828891 10.1126/science.1241165 PMC3807819

[embr202255664-bib-0182] Soderholm AT , Pedicord VA (2019) Intestinal epithelial cells: at the interface of the microbiota and mucosal immunity. Immunology 158: 267–280 31509239 10.1111/imm.13117 PMC6856932

[embr202255664-bib-0183] Somm E , Henry H , Bruce SJ , Aeby S , Rosikiewicz M , Sykiotis GP , Asrih M , Jornayvaz FR , Denechaud PD , Albrecht U *et al* (2017) β‐Klotho deficiency protects against obesity through a crosstalk between liver, microbiota, and brown adipose tissue. JCI Insight 2: e91809 28422755 10.1172/jci.insight.91809 PMC5396514

[embr202255664-bib-0184] Soret R , Chevalier J , De Coppet P , Poupeau G , Derkinderen P , Segain JP , Neunlist M (2010) Short‐chain fatty acids regulate the enteric neurons and control gastrointestinal motility in rats. Gastroenterology 138: 1772–1782 20152836 10.1053/j.gastro.2010.01.053

[embr202255664-bib-0185] Sorribas M , Jakob MO , Yilmaz B , Li H , Stutz D , Noser Y , de Gottardi A , Moghadamrad S , Hassan M , Albillos A *et al* (2019) FXR modulates the gut‐vascular barrier by regulating the entry sites for bacterial translocation in experimental cirrhosis. J Hepatol 71: 1126–1140 31295531 10.1016/j.jhep.2019.06.017

[embr202255664-bib-0186] Spadoni I , Zagato E , Bertocchi A , Paolinelli R , Hot E , Di Sabatino A , Caprioli F , Bottiglieri L , Oldani A , Viale G *et al* (2015) A gut‐vascular barrier controls the systemic dissemination of bacteria. Science 350: 830–834 26564856 10.1126/science.aad0135

[embr202255664-bib-0187] Suez J , Zmora N , Segal E , Elinav E (2019) The pros, cons, and many unknowns of probiotics. Nat Med 25: 716–729 31061539 10.1038/s41591-019-0439-x

[embr202255664-bib-0188] Suez J , Zmora N , Zilberman‐Schapira G , Mor U , Dori‐Bachash M , Bashiardes S , Zur M , Regev‐Lehavi D , Ben‐Zeev Brik R , Federici S *et al* (2018) Post‐antibiotic gut mucosal microbiome reconstitution is impaired by probiotics and improved by autologous FMT. Cell 174: 1406–1423 30193113 10.1016/j.cell.2018.08.047

[embr202255664-bib-0189] Suh SH , Choe K , Hong SP , Jeong S‐H , Mäkinen T , Kim KS , Alitalo K , Surh CD , Koh GY , Song J‐H (2019) Gut microbiota regulates lacteal integrity by inducing VEGF‐C in intestinal villus macrophages. EMBO Rep 20: e46927 30783017 10.15252/embr.201846927 PMC6446200

[embr202255664-bib-0190] Sun J , Furio L , Mecheri R , van der Does AM , Lundeberg E , Saveanu L , Chen Y , van Endert P , Agerberth B , Diana J (2015) Pancreatic β‐cells limit autoimmune diabetes via an immunoregulatory antimicrobial peptide expressed under the influence of the gut microbiota. Immunity 43: 304–317 26253786 10.1016/j.immuni.2015.07.013

[embr202255664-bib-0191] Sun L , Xie C , Wang G , Wu Y , Wu Q , Wang X , Liu J , Deng Y , Xia J , Chen B *et al* (2018) Gut microbiota and intestinal FXR mediate the clinical benefits of metformin. Nat Med 24: 1919–1929 30397356 10.1038/s41591-018-0222-4 PMC6479226

[embr202255664-bib-0192] Suzuki T , Hara H (2009) Quercetin enhances intestinal barrier function through the assembly of zonnula Occludens‐2, Occludin, and Claudin‐1 and the expression of Claudin‐4 in Caco‐2 cells. J Nutr 139: 965–974 19297429 10.3945/jn.108.100867

[embr202255664-bib-0193] Swann JR , Want EJ , Geier FM , Spagou K , Wilson ID , Sidaway JE , Nicholson JK & Holmes E (2011) Systemic gut microbial modulation of bile acid metabolism in host tissue compartments. Proc Natl Acad Sci U S A 108 Suppl 1: 4523–4530 20837534 10.1073/pnas.1006734107 PMC3063584

[embr202255664-bib-0194] Tachedjian G , Aldunate M , Bradshaw CS , Cone RA (2017) The role of lactic acid production by probiotic *Lactobacillus* species in vaginal health. Res Microbiol 168: 782–792 28435139 10.1016/j.resmic.2017.04.001

[embr202255664-bib-0195] Tan C , Wu Q , Wang H , Gao X , Xu R , Cui Z , Zhu J , Zeng X , Zhou H , He Y *et al* (2021) Dysbiosis of gut microbiota and short‐chain fatty acids in acute ischemic stroke and the subsequent risk for poor functional outcomes. JPEN J Parenter Enteral Nutr 45: 518–529 32473086 10.1002/jpen.1861 PMC8048557

[embr202255664-bib-0196] Tan X , Liu Y , Long J , Chen S , Liao G , Wu S , Li C , Wang L , Ling W , Zhu H (2019) Trimethylamine N‐Oxide aggravates liver steatosis through modulation of bile acid metabolism and inhibition of farnesoid X receptor signaling in nonalcoholic fatty liver disease. Mol Nutr Food Res 63: e1900257 31095863 10.1002/mnfr.201900257

[embr202255664-bib-0197] Thaiss CA , Itav S , Rothschild D , Meijer MT , Levy M , Moresi C , Dohnalová L , Braverman S , Rozin S , Malitsky S *et al* (2016a) Persistent microbiome alterations modulate the rate of post‐dieting weight regain. Nature 540: 544–551 27906159 10.1038/nature20796

[embr202255664-bib-0198] Thaiss CA , Levy M , Korem T , Dohnalová L , Shapiro H , Jaitin DA , David E , Winter DR , Gury‐BenAri M , Tatirovsky E *et al* (2016b) Microbiota diurnal rhythmicity programs host transcriptome oscillations. Cell 167: 1495–1510 27912059 10.1016/j.cell.2016.11.003

[embr202255664-bib-0199] Tierney BT , Yang Z , Luber JM , Beaudin M , Wibowo MC , Baek C , Mehlenbacher E , Patel CJ , Kostic AD (2019) The landscape of genetic content in the gut and oral human microbiome. Cell Host Microbe 26: 283–295 31415755 10.1016/j.chom.2019.07.008 PMC6716383

[embr202255664-bib-0200] Tilg H , Zmora N , Adolph TE , Elinav E (2020) The intestinal microbiota fuelling metabolic inflammation. Nat Rev Immunol 20: 40–54 31388093 10.1038/s41577-019-0198-4

[embr202255664-bib-0201] Tobias DK , Lawler PR , Harada PH , Demler OV , Ridker PM , Manson JE , Cheng S , Mora S (2018) Circulating branched‐chain amino acids and incident cardiovascular disease in a prospective cohort of US women. Circ Genom Precis Med 11: e002157 29572205 10.1161/CIRCGEN.118.002157 PMC5880282

[embr202255664-bib-0202] Tough IR , Forbes S , Tolhurst R , Ellis M , Herzog H , Bornstein JC , Cox HM (2011) Endogenous peptide YY and neuropeptide Y inhibit colonic ion transport, contractility and transit differentially via Y₁ and Y₂ receptors. Br J Pharmacol 164: 471–484 21457230 10.1111/j.1476-5381.2011.01401.x PMC3188896

[embr202255664-bib-0203] Trabelsi M‐S , Daoudi M , Prawitt J , Ducastel S , Touche V , Sayin SI , Perino A , Brighton CA , Sebti Y , Kluza J *et al* (2015) Farnesoid X receptor inhibits glucagon‐like peptide‐1 production by enteroendocrine L cells. Nat Commun 6: 7629 26134028 10.1038/ncomms8629 PMC4579574

[embr202255664-bib-0204] Tremaroli V , Karlsson F , Werling M , Ståhlman M , Kovatcheva‐Datchary P , Olbers T , Fändriks L , le Roux CW , Nielsen J , Bäckhed F (2015) Roux‐en‐Y gastric bypass and vertical banded gastroplasty induce long‐term changes on the human gut microbiome contributing to fat mass regulation. Cell Metab 22: 228–238 26244932 10.1016/j.cmet.2015.07.009 PMC4537510

[embr202255664-bib-0205] Trompette A , Gollwitzer ES , Yadava K , Sichelstiel AK , Sprenger N , Ngom‐Bru C , Blanchard C , Junt T , Nicod LP , Harris NL *et al* (2014) Gut microbiota metabolism of dietary fiber influences allergic airway disease and hematopoiesis. Nat Med 20: 159–166 24390308 10.1038/nm.3444

[embr202255664-bib-0206] Turnbaugh PJ , Hamady M , Yatsunenko T , Cantarel BL , Duncan A , Ley RE , Sogin ML , Jones WJ , Roe BA , Affourtit JP *et al* (2009) A core gut microbiome in obese and lean twins. Nature 457: 480–484 19043404 10.1038/nature07540 PMC2677729

[embr202255664-bib-0207] Turnbaugh PJ , Ley RE , Mahowald MA , Magrini V , Mardis ER , Gordon JI (2006) An obesity‐associated gut microbiome with increased capacity for energy harvest. Nature 444: 1027–1031 17183312 10.1038/nature05414

[embr202255664-bib-0208] Vaishnava S , Behrendt CL , Ismail AS , Eckmann L , Hooper LV (2008) Paneth cells directly sense gut commensals and maintain homeostasis at the intestinal host‐microbial interface. Proc Natl Acad Sci U S A 105: 20858–20863 19075245 10.1073/pnas.0808723105 PMC2603261

[embr202255664-bib-0209] Velazquez‐Villegas LA , Perino A , Lemos V , Zietak M , Nomura M , Pols TWH , Schoonjans K (2018) TGR5 signalling promotes mitochondrial fission and beige remodelling of white adipose tissue. Nat Commun 9: 245 29339725 10.1038/s41467-017-02068-0 PMC5770450

[embr202255664-bib-0210] Venkatesh M , Mukherjee S , Wang H , Li H , Sun K , Benechet AP , Qiu Z , Maher L , Redinbo MR , Phillips RS *et al* (2014) Symbiotic bacterial metabolites regulate gastrointestinal barrier function via the xenobiotic sensor PXR and Toll‐like receptor 4. Immunity 41: 296–310 25065623 10.1016/j.immuni.2014.06.014 PMC4142105

[embr202255664-bib-0211] Vrzáčková N , Ruml T , Zelenka J (2021) Postbiotics, metabolic signaling, and cancer. Molecules 26: 1528 33799580 10.3390/molecules26061528 PMC8000401

[embr202255664-bib-0212] Wahlström A , Sayin SI , Marschall H‐U , Bäckhed F (2016) Intestinal crosstalk between bile acids and microbiota and its impact on host metabolism. Cell Metab 24: 41–50 27320064 10.1016/j.cmet.2016.05.005

[embr202255664-bib-0213] Wang L , Zhu Q , Lu A , Liu X , Zhang L , Xu C , Liu X , Li H , Yang T (2017) Sodium butyrate suppresses angiotensin II‐induced hypertension by inhibition of renal (pro)renin receptor and intrarenal renin–angiotensin system. J Hypertens 35: 1899 28509726 10.1097/HJH.0000000000001378 PMC11157961

[embr202255664-bib-0214] Wang RX , Lee JS , Campbell EL , Colgan SP (2020) Microbiota‐derived butyrate dynamically regulates intestinal homeostasis through regulation of actin‐associated protein synaptopodin. Proc Natl Acad Sci U S A 117: 11648–11657 32398370 10.1073/pnas.1917597117 PMC7260972

[embr202255664-bib-0215] Wang S , Dong W , Liu L , Xu M , Wang Y , Liu T , Zhang Y , Wang B , Cao H (2019) Interplay between bile acids and the gut microbiota promotes intestinal carcinogenesis. Mol Carcinog 58: 1155–1167 30828892 10.1002/mc.22999 PMC6593857

[embr202255664-bib-0216] Watanabe M , Houten SM , Mataki C , Christoffolete MA , Kim BW , Sato H , Messaddeq N , Harney JW , Ezaki O , Kodama T *et al* (2006) Bile acids induce energy expenditure by promoting intracellular thyroid hormone activation. Nature 439: 484–489 16400329 10.1038/nature04330

[embr202255664-bib-0217] Weitkunat K , Schumann S , Nickel D , Kappo KA , Petzke KJ , Kipp AP , Blaut M , Klaus S (2016) Importance of propionate for the repression of hepatic lipogenesis and improvement of insulin sensitivity in high‐fat diet‐induced obesity. Mol Nutr Food Res 60: 2611–2621 27467905 10.1002/mnfr.201600305 PMC5215627

[embr202255664-bib-0218] Weitkunat K , Stuhlmann C , Postel A , Rumberger S , Fankhänel M , Woting A , Petzke KJ , Gohlke S , Schulz TJ , Blaut M *et al* (2017) Short‐chain fatty acids and inulin, but not guar gum, prevent diet‐induced obesity and insulin resistance through differential mechanisms in mice. Sci Rep 7: 6109 28733671 10.1038/s41598-017-06447-x PMC5522422

[embr202255664-bib-0219] Wilck N , Matus MG , Kearney SM , Olesen SW , Forslund K , Bartolomaeus H , Haase S , Mähler A , Balogh A , Markó L *et al* (2017) Salt‐responsive gut commensal modulates TH17 axis and disease. Nature 551: 585–589 29143823 10.1038/nature24628 PMC6070150

[embr202255664-bib-0220] Worthmann A , John C , Rühlemann MC , Baguhl M , Heinsen F‐A , Schaltenberg N , Heine M , Schlein C , Evangelakos I , Mineo C *et al* (2017) Cold‐induced conversion of cholesterol to bile acids in mice shapes the gut microbiome and promotes adaptive thermogenesis. Nat Med 23: 839–849 28604703 10.1038/nm.4357

[embr202255664-bib-0221] Wu Y , Ma N , Song P , He T , Levesque C , Bai Y , Zhang A , Ma X (2019) Grape seed proanthocyanidin affects lipid metabolism via changing gut microflora and enhancing propionate production in weaned pigs. J Nutr 149: 1523–1532 31175811 10.1093/jn/nxz102

[embr202255664-bib-0222] Xiong Y , Miyamoto N , Shibata K , Valasek MA , Motoike T , Kedzierski RM , Yanagisawa M (2004) Short‐chain fatty acids stimulate leptin production in adipocytes through the G protein‐coupled receptor GPR41. Proc Natl Acad Sci U S A 101: 1045–1050 14722361 10.1073/pnas.2637002100 PMC327148

[embr202255664-bib-0223] Yajima T , Inoue R , Matsumoto M , Yajima M (2011) Non‐neuronal release of ACh plays a key role in secretory response to luminal propionate in rat colon. J Physiol 589: 953–962 21135046 10.1113/jphysiol.2010.199976 PMC3060372

[embr202255664-bib-0224] Yamashita H , Maruta H , Jozuka M , Kimura R , Iwabuchi H , Yamato M , Saito T , Fujisawa K , Takahashi Y , Kimoto M *et al* (2009) Effects of acetate on lipid metabolism in muscles and adipose tissues of type 2 diabetic Otsuka Long‐Evans Tokushima Fatty (OLETF) rats. Biosci Biotechnol Biochem 73: 570–576 19270372 10.1271/bbb.80634

[embr202255664-bib-0225] Yang W , Yu T , Huang X , Bilotta AJ , Xu L , Lu Y , Sun J , Pan F , Zhou J , Zhang W *et al* (2020) Intestinal microbiota‐derived short‐chain fatty acids regulation of immune cell IL‐22 production and gut immunity. Nat Commun 11: 4457 32901017 10.1038/s41467-020-18262-6 PMC7478978

[embr202255664-bib-0226] Yatsunenko T , Rey FE , Manary MJ , Trehan I , Dominguez‐Bello MG , Contreras M , Magris M , Hidalgo G , Baldassano RN , Anokhin AP *et al* (2012) Human gut microbiome viewed across age and geography. Nature 486: 222–227 22699611 10.1038/nature11053 PMC3376388

[embr202255664-bib-0227] Yu J , Luo Y , Zhu Z , Zhou Y , Sun L , Gao J , Sun J , Wang G , Yao X , Li W (2019) A tryptophan metabolite of the skin microbiota attenuates inflammation in patients with atopic dermatitis through the aryl hydrocarbon receptor. J Allergy Clin Immunol 143: 2108–2119 30578876 10.1016/j.jaci.2018.11.036

[embr202255664-bib-0228] Zaibi MS , Stocker CJ , O'Dowd J , Davies A , Bellahcene M , Cawthorne MA , Brown AJH , Smith DM , Arch JRS (2010) Roles of GPR41 and GPR43 in leptin secretory responses of murine adipocytes to short chain fatty acids. FEBS Lett 584: 2381–2386 20399779 10.1016/j.febslet.2010.04.027

[embr202255664-bib-0229] Zaiss MM , Rapin A , Lebon L , Dubey LK , Mosconi I , Sarter K , Piersigilli A , Menin L , Walker AW , Rougemont J *et al* (2015) The intestinal microbiota contributes to the ability of helminths to modulate allergic inflammation. Immunity 43: 998–1010 26522986 10.1016/j.immuni.2015.09.012 PMC4658337

[embr202255664-bib-0230] Zaki MH , Boyd KL , Vogel P , Kastan MB , Lamkanfi M , Kanneganti T‐D (2010) The NLRP3 inflammasome protects against loss of epithelial integrity and mortality during experimental colitis. Immunity 32: 379–391 20303296 10.1016/j.immuni.2010.03.003 PMC2982187

[embr202255664-bib-0231] Zeevi D , Korem T , Zmora N , Israeli D , Rothschild D , Weinberger A , Ben‐Yacov O , Lador D , Avnit‐Sagi T , Lotan‐Pompan M *et al* (2015) Personalized nutrition by prediction of glycemic responses. Cell 163: 1079–1094 26590418 10.1016/j.cell.2015.11.001

[embr202255664-bib-0232] Zelante T , Iannitti RG , Cunha C , De Luca A , Giovannini G , Pieraccini G , Zecchi R , D'Angelo C , Massi‐Benedetti C , Fallarino F *et al* (2013) Tryptophan catabolites from microbiota engage aryl hydrocarbon receptor and balance mucosal reactivity via interleukin‐22. Immunity 39: 372–385 23973224 10.1016/j.immuni.2013.08.003

[embr202255664-bib-0233] Zhang S , Zhang S , Hu L , Zhai L , Xue R , Ye J , Chen L , Cheng G , Mruk J , Kunapuli SP *et al* (2015) Nucleotide‐binding oligomerization domain 2 receptor is expressed in platelets and enhances platelet activation and thrombosis. Circulation 131: 1160–1170 25825396 10.1161/CIRCULATIONAHA.114.013743 PMC4382913

[embr202255664-bib-0234] Zhang Z , Mocanu V , Cai C , Dang J , Slater L , Deehan EC , Walter J , Madsen KL (2019) Impact of fecal microbiota transplantation on obesity and metabolic syndrome—a systematic review. Nutrients 11: 2291 31557953 10.3390/nu11102291 PMC6835402

[embr202255664-bib-0235] Zhao Y , Chen F , Wu W , Sun M , Bilotta AJ , Yao S , Xiao Y , Huang X , Eaves‐Pyles TD , Golovko G *et al* (2018) GPR43 mediates microbiota metabolite SCFA regulation of antimicrobial peptide expression in intestinal epithelial cells via activation of mTOR and STAT3. Mucosal Immunol 11: 752–762 29411774 10.1038/mi.2017.118 PMC5976519

[embr202255664-bib-0236] Zheng X , Cai X , Hao H (2022) Emerging targetome and signalome landscape of gut microbial metabolites. Cell Metab 34: 35–58 34986337 10.1016/j.cmet.2021.12.011

[embr202255664-bib-0237] Zhu H , Cao C , Wu Z , Zhang H , Sun Z , Wang M , Xu H , Zhao Z , Wang Y , Pei G *et al* (2021) The probiotic *L. casei* Zhang slows the progression of acute and chronic kidney disease. Cell Metab 33: 1926–1942 34270930 10.1016/j.cmet.2021.06.014

[embr202255664-bib-0238] Zhu W , Gregory JC , Org E , Buffa JA , Gupta N , Wang Z , Li L , Fu X , Wu Y , Mehrabian M *et al* (2016) Gut microbial metabolite TMAO enhances platelet hyperreactivity and thrombosis risk. Cell 165: 111–124 26972052 10.1016/j.cell.2016.02.011 PMC4862743

[embr202255664-bib-0239] Ziętak M , Kovatcheva‐Datchary P , Markiewicz LH , Ståhlman M , Kozak LP , Bäckhed F (2016) Altered microbiota contributes to reduced diet‐induced obesity upon cold exposure. Cell Metab 23: 1216–1223 27304513 10.1016/j.cmet.2016.05.001 PMC4911343

[embr202255664-bib-0240] Zmora N , Suez J , Elinav E (2018) You are what you eat: diet, health and the gut microbiota. Nat Rev Gastroenterol Hepatol 16: 35–56 10.1038/s41575-018-0061-2 30262901

